# A Malonyl-Based Scaffold for Conjugatable Multivalent Carbohydrate-BODIPY Presentations

**DOI:** 10.3390/molecules24112050

**Published:** 2019-05-29

**Authors:** Clara Uriel, Rebeca Sola-Llano, Jorge Bañuelos, Ana M. Gomez, J. Cristobal Lopez

**Affiliations:** 1 Instituto de Química Orgánica General, Consejo Superior de Investigaciones Científicas (IQOG-CSIC), 28006 Madrid, Spain; ana.gomez@csic.es; 2 Departamento Química Física, Universidad del País Vasco (UPV/EHU), 48080 Bilbao, Spain; rebeca.sola@ehu.es (R.S.-L.); jorge.banuelos@ehu.es (J.B.)

**Keywords:** tetravalent scaffold, BODIPY, conjugation, glycosylation, monosaccharide, multivalent presentation

## Abstract

A concise synthetic route from methylmalonate to a tetravalent aliphatic scaffold has been developed. The ensuing tetra-tethered derivative is equipped with two hydroxyl groups, as well as orthogonal alkene and alkyne functionalities. The usefulness of the scaffold has been demonstrated with the preparation of two representative multivalent derivatives: (i) a tetravalent compound containing two D-mannose units, one fluorescent boron-dipyrromethene (BODIPY) dye and a suitably functionalized amino acid and (ii) by way of dimerization and saponification, a water-soluble tetramannan derivative containing two fluorescent BODIPY units. Additionally, photophysical measurements conducted on these derivatives support the viability of the herein designed single and double BODIPY-labeled carbohydrate-based clusters as fluorescent markers.

## 1. Introduction

It is currently accepted that a broad range of biological functions is associated with recognition events between carbohydrates and proteins [1,2]. On the other hand, advances in chemical biology and biomedicine have benefitted from the emergence of small-molecule fluorescent probes [3,4] in combination with fluorescence spectroscopy or microscopy detection techniques [5,6,7,8,9,10]. Recently, encompassing these two research areas, several examples on the usefulness of fluorescently labeled carbohydrates in the investigation of biological processes have appeared in the literature [11,12,13,14]. In a complementary manner, it has been shown that a glycosyl moiety attached to a fluorescent probe might play a key role as a targeting [15,16,17,18] and internalizing agent for the probe [10,11,12,13,14,15,16,17,18,19,20,21,22], sometimes even providing less cytotoxic entities [23,24].

A further refinement related to the aforementioned carbohydrate-protein interactions came with the realization that individual carbohydrate-ligands bind to a protein site weakly, and therefore the high levels of recognition in biological events had to be associated to the existence of the so-called multivalent interactions [25]. In these (multivalent) interactions, multiple copies of the ligands and receptors must be involved in the binding event in order to provide the significantly high levels of specificity required for the biological processes to take place [26,27,28]. Accordingly, the quest for multivalent carbohydrate ligands has led to the design of several types of molecular scaffolds, for example, aliphatic, aromatic, carbohydrate, and peptide scaffolds [29,30,31,32,33,34,35,36], with the ability to lodge multiple copies of sugar epitopes [37]. Furthermore, glycan-coated polymers and other biomaterials have also been used in the multivalent presentation of sugar epitopes and shown their usefulness in lectin-mediated interactions [38].

**Figure 1:**
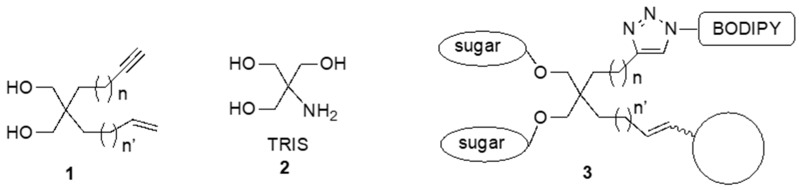
Tetravalent scaffold (**1**), Lee’s TRIS aliphatic scaffold (**2**), and target molecules (**3**) (this work). BODIPY: boron-dipyrromethene

We have recently become interested in the preparation of novel fluorescent conjugatable fluorophores [39,40], in particular, boron-dipyrromethene (BODIPY) [41,42] dyes, which in combination with carbohydrates could lead to water-soluble fluorophores [43,44,45] with potential biological interest [46,47]. In this communication, we report a novel strategy towards a tetravalent scaffold (**1**, Figure 1), which enables the multivalent presentation of saccharide derivatives in conjunction with a BODIPY fluorophore.

According to our design, the aliphatic scaffold **1**, reminiscent of Lee’s AB_3_, tris(hydroxymethyl) aminomethane (**2**, TRIS) [48] (Figure 1), is easily available from ethyl malonate. It contains two primary hydroxyl groups for glycosylation and two additional (orthogonal) sites, an alkyne and an alkene for further functionalization. α-d-Mannose was chosen to be introduced in the scaffold as a relevant ligand that is recognized by macrophage mannose receptors [49], microbacterial membranes [50], as well as by lectins, such as DC-SIGN [51,52], and concanavalin A [31,33,36]. As a consequence, α-d-Mannose has been incorporated into a broad range of multivalent structures [20,30,53]. The incorporation of the BODIPY chromophore to the scaffold could be achieved by copper(I)-catalyzed azide-alkyne cycloaddition (CuAAC) [54,55], whereas the remaining terminal olefin could be used for further conjugation to olefin-containing (bio)molecules of interest (e.g., amino acids, peptides, nucleic acids, etc.) by a cross-metathesis reaction [56,57], leading to mannosyl BODIPY-labeled species type **3** (Figure 1).

## 2. Results and Discussion

### 2.1. Synthesis, Glycosylation, and CuAAC Reaction of The Scaffold

**Scheme 1:**
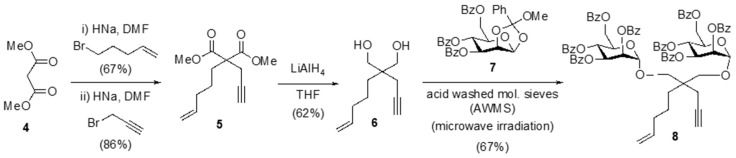
Synthesis of dimannoside (**8**) from methylmalonate (**4**).

Based on the well-studied chemistry of malonate diesters [58], the sequential bis-alkylation of dimethyl malonate (**4**) with pentenyl bromide and propargyl bromide paved the way to, previously unreported, ene-yne derivative **5** (Scheme 1). Subsequent LiAlH_4_-mediated reduction of **5** led to diol **6**, which was then glycosylated with readily prepared methyl orthoester (MeOE) **7** [59,60] (acid washed molecular sieves (AWMS), microwave irradiation) to furnish dimannan **8** in acceptable yield (Scheme 1) [60].

Pseudodisaccharide **8** possesses two orthogonal anchoring sites for further conjugation, and accordingly, the copper-catalyzed azide-alkyne cycloaddition (CuAAC) reaction of **8** with azidomethyl boron-dipyrromethene (BODIPY) derivative **9** [39] was carried out to furnish carbohydrate-BODIPY hybrid **10**, leaving the terminal olefin ready for cross-metathesis reactions [56,57].

### 2.2. Cross-Metathesis Reactions

As proof of concept on the use of **10** in conjugation protocols, we carried out the cross-metathesis reaction of **10** and commercially available Boc-protected *O*-allyl-l-tyrosine (**11**), as an example of a functionalized molecule, to yield tetravalent derivative **12** (68% yield). Likewise, dimerization of **10** by cross-metathesis took place smoothly to yield compound **13**, containing two BODIPY units and four copies of carbohydrate ligands (44% yield, 79% corrected yield based on recovered, unreacted, olefin **10**).

### 2.3. De-O-Benzoylation of Dimer **13**

**Figure 2:**
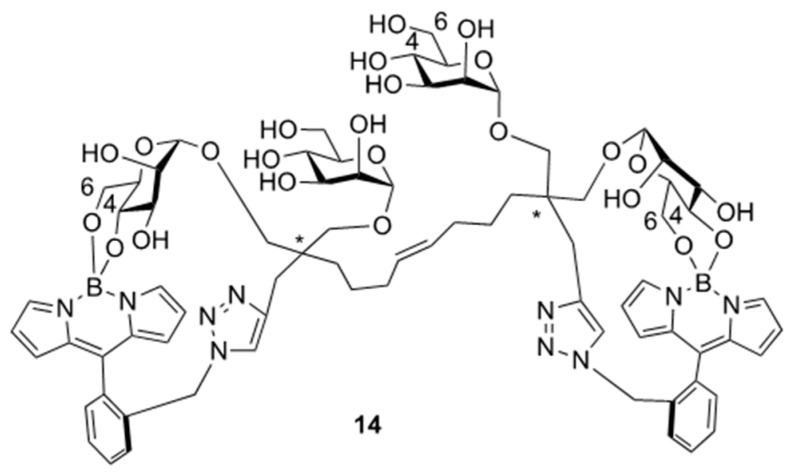
Fluorescently-labeled tetramannan derivative (**14**).

Finally, de-O-benzoylation of dimeric structure **13** (NaOMe, MeOH, room temperature) yielded fluorescent, water-soluble, tetramannan compound **14** (Figure 2), as a diastereomeric mixture, wherein the fluorine atoms at boron, in the BODIPY cores, had been replaced by the oxygen atoms at C-4 and C-6 in the mannose units. The structural assignment of compound **14** was unambiguously made based on (i) the absence of the fluorine signals in its ^19^F-NMR spectrum (Supplementary materials, Figure S26), (ii) its mass spectrum, and (iii) the observed chemical shift (1.45 ppm) in its ^11^B-NMR spectrum (Supplementary materials, Figure S25). The latter allowed to unequivocally assign the BODIPY units as bounded to a 1,3-diol (^11^B-NMR, chemical shift range from 0.5 to 1.9 ppm) rather than to a *cis*-1,2-diol (^11^B-NMR, chemical shift range from 4.0 to 5.9 ppm), according to previous studies in related compounds [61]. This reaction, although somehow unexpected, provided 4,4′-dialkoxy BODIPY derivative **14** endowed with a respectable fluorescence quantum yield (ϕ = 0.30) in water.

### 2.4. Photophysical Studies

**Figure 3:**
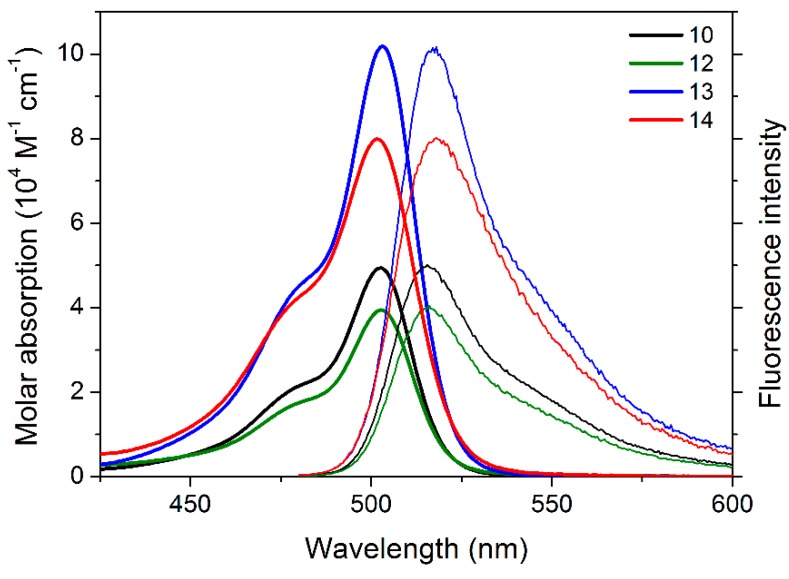
Absorption (bold line) and normalized fluorescence (thin line) spectra of boron-dipyrromethene (BODIPY)-labeled tetravalent (**10** and **12)** and hexavalent (**13**) derivatives in diluted solutions (2 μM) of EtOAc. The corresponding spectra of compound **14** in water is also included.

The photophysical signatures of the single BODIPY-labeled carbohydrate-based scaffolds (compounds **10** and **12**, Table 1) retain the characteristics of the isolated BODIPY chromophoric core [62] and 8-*ortho*-benzyl BODIPYs [40]. The linkage of the BODIPY to the tetravalent cluster through the *ortho* position of the chromophoric 8-phenyl induces the required sterical hindrance to vanish the impact of such phenyl substituent into the photophysics of the BODIPY core. Thus, as a result of such constrained geometry, the 8-phenyl substituent is disposed of almost orthogonally with respect to the dipyrrin backbone and electronically decoupled, thereby, the spectral bands’ positions and profiles are unaltered by the attachment of the 8-aryl group (Figure 3). Moreover, such hindered 8-phenyl group is placed at a fixed position, hence, avoiding non-radiative deactivation channels associated to its free motion [63] and ensuring a bright fluorescence response (up to 90% for **10**, Table 1) and long lifetimes (around 5–7 ns in Table 1). Note that the overall photophysical properties of both dyes **10** and **12** are very similar. Therefore, the size and length of the functionalization attached to the *ortho* position of the 8-phenyl substituent has a low impact on the photophysics of the photoactive BODIPY unit, albeit the presence of the electron donor terminal amino acid seems to slightly decrease the fluorescence efficiency (Table 1).

**Scheme 2:**
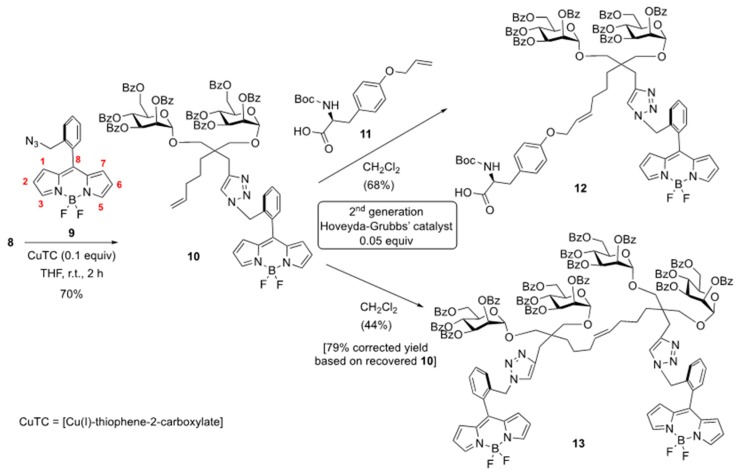
Synthesis of tetravalent and hexavalent derivatives (**12**) and (**13**).

Compound **13** features two chromophoric units tethered to the carbohydrate-based cluster (Scheme 2). The position and shape of the absorption spectrum of dye **13** matches that of its precursor **10**, but the intensity shows a two-fold enhancement (up to 10^5^ M^−1^ cm^−1^, Figure 3) according to the presence of two BODIPYs in the cluster. Therefore, each chromophoric subunit contributes additively to the whole spectral transition of the molecular assembly. Furthermore, the fluorescence position also remains the same, and the fluorescence quantum yields and lifetimes are noticeable (higher than 50% and around 5–6 ns, respectively, Table 1), in spite of the large size of the molecule (up to four carbohydrates and two BODIPYs covalently linked in a single structure) and the conformational flexibility of the spacers. On the other hand, the absence of any change in the spectral profile of compound **13** rules out any intramolecular interaction between the two BODIPY units (such as exciton coupling) because they are most certainly disposed of far away from each other. In line with these results, de-*O*-benzoylated compound **14** (with Boron-oxygen substitution) also displays strong spectral bands even in water (Figure 3) with a notable fluorescence response (30%, Table 1). Such fluorescence efficiency is lower than that observed in the fluorinated derivative **13**, owing to the known increase of the non-radiative pathways upon the presence of alkoxy groups replacing fluorine atoms at the boron bridge [64]. Nevertheless, the herein reported fluorescence efficiency in water is higher than that reported for the structurally related “BODIPY-labeled” carbohydrates via the boron center [61], owing to the imposed geometrical constraints around the key chromophoric C-8-position of the BODIPY core.

Summing up, an efficiently accessible tetravalent malonyl-derived scaffold allows the suitable incorporation of two (or four) carbohydrate units, tagged with one (or two) fluorescent BODIPY label(s). Variations in the carbohydrate ligands and/or the BODIPY unit(s) could provide a range of fluorescent glycoprobes.

## 3. Experimental Section

### 3.1. Chemistry

#### 3.1.1. General Procedure for Alkylation

To a suspension of NaH (60% in mineral oil, 1.2 eq.) in anhydrous DMF under Ar atmosphere at 0 °C, the corresponding malonate (1 eq.) was added slowly, and the mixture was stirred for 15 min. Then, the alkyl bromide (1 eq.) was added dropwise, and the mixture was allowed to react at room temperature (r.t.) during 24 h. The solution was diluted with Et_2_O:Toluene (8:2) and washed with saturated ammonium chloride solution. The aqueous phase was extracted with Et_2_O. The combined organic layers were dried over NaSO_4_, filtered, and concentrated. The crude product was purified by flash chromatography on silica gel.

*Dimethyl 2-(pent-4′-enyl)-2-(prop-2′-ynyl) malonate (**5**):* Following the general alkylation procedure, dimethyl malonate (2 mL, 17.4 mmol) was added dropwise to a suspension of NaH (840 mg, 20.9 mmol) in anhydrous DMF (10 mL); after 15 min, 5-bromopent-1-ene (2.1 mL, 17.4 mmol) was added, and the mixture was stirred during 24 h, quenched, and purified by flash chromatography (hexane:EtOAc, 7:3) to afford dimethyl 2-(pent-4′-enyl) malonate 2.36 g (67%) [65]. This derivative (2.36 g, 11.8 mmol) was subsequently added to a suspension of NaH (571 mg, 14.2 mmol) in anhydrous DMF (10 mL). After 15 min, propargyl bromide (1.07 mL, 14.2 mmol) was slowly added, and the mixture was stirred for 24 h. The reaction was quenched and purified by flash chromatography (hexane: EtOAc, 95:5) to give compound **5**, 2.41 g (86%). ^1^H-NMR (400 MHz, CDCl_3_): 1.20–1.32 (m, 2H), 2.00 (t, *J* =2.7 Hz, 1H), 2.02–2.09 (m, 1H), 2.82 (d, *J* = 2.7 Hz, 2H), 3.73 (s, 6H), 4.93–5.05 (m, 1H), 5.77 (ddt, *J* = 16.8, 10.2, 6.6 Hz, 1-H). ^13^C-NMR (100 MHz; CDCl_3_): 22.8, 23.2, 31.5, 33.6, 52.7 (×2), 56.8, 71.2, 78.7, 115.0, 137.8, 170.6 (×2). HRMS (ESI-QqTOF) *m*/*z*: Calc. for C_13_H_19_O_4_ [M+H]^+^ 239.1283, found 239.1285.

*2-(pent-4′-enyl)-2-(prop-2′-ynyl)propane-1,3-diol (**6**)*: A solution of compound **5** (266 mg, 1 mmol) in anhydrous THF (5 mL) was added dropwise to a suspension of LiAlH_4_ (95 mg, 2.5 mmol) in THF (5 mL) at 0 °C. The resulting mixture was stirred at r.t. for 2 h. The reaction was quenched by slow addition of a saturated solution of Na_2_SO_4_ (10 mL) and stirring the mixture for 15 min. The resulting white slurry was filtered through celite and washed with Et_2_O (2 × 15 mL). The filtrate was concentrated and purified by flash chromatography (hexane:EtOAc 1:1) to give **6** (113 mg, 62 %). ^1^H-NMR (400 MHz; CDCl_3_): 1.32–1.33 (m, 2H), 1.99 (t, *J* =2.7 Hz, 1H), 2.02–2.04 (m, 1H), 2.24 (d, *J* = 2.7 Hz, 2H), 3.52–3.68 (m, 4H), 4.92–5.02 (m, 1H), 5.78 (ddt, *J* = 16.9, 10.2, 6.7 Hz, 1H). ^13^C-NMR (100 MHz; CDCl_3_): 21.3, 22.3, 31.0, 34.4, 41.6, 67.6, 70.7, 81.1, 114.8, 138.6. HRMS (ESI-QqTOF) *m*/*z*: Calc. for C_11_H_19_O_2_ [M+H]^+^ 182.13071, found 182.13231.

*Compound **8**:* A mixture of methyl orthoester MeOE 7 [61] (305 mg, 0.5 mmol) and compound **6** (36.4 mg, 0.2 mmol) in toluene (5 mL) was azeotroped to dryness, and subsequently kept overnight under high vacuum. This mixture was then dissolved in dry CH_2_Cl_2_ (8 mL), and acid-washed molecular sieves AW-300 (Sigma-Aldrich, Saint Louis, MI, USA) (1.6 mm pellets, 3 g) were added. The mixture was stirred at 50 °C and 200 W during 2 h [61]. Then, the crude was filtered and purified by flash chromatography (Hexane: EtOAc, 7:3) to give **8** (179 mg, 67%). ^1^H-NMR (500 MHz; CDCl_3_): 8.12–7.76 (m, 15H), 7.59–7.19 (m, 25H), 6.14 (t, *J* = 10.0 Hz, 1H), 6.13 (t, *J* = 10.0 Hz, 1H), 5.93–5.83 (m, 3H), 5.75–5.73 (m, 2H), 5.17 (d, *J* = 2.1 Hz, 1H), 5.16 (d, *J* = 2.1 Hz, 1H), 5.16 (d, *J* = 2.0 Hz, 1H), 5.12–5.00 (m, 2H), 4.78–4.73 (m, 2H), 4.56–4.48 (m,4H), 3.91 (d, *J* = 9.7 Hz, 2H), 3.57 (d, *J* = 9.7 Hz, 2H), 2.53 (dd, *J* = 16.9, 2.7 Hz, 1H), 2.40 (dd, *J* = 16.9, 2.7 Hz, 1H), 2.21–2.12 (m, 2H), 2.06 (t, *J* = 2.6 Hz, 1H), 1.68–1.57 (m, 2H), 1.52–1.43 (m, 2H). ^13^C-NMR (125 MHz; CDCl_3_): 166.3 (×2), 165.6 (×4), 165.4 (×2), 138.4, 133.5 (×2), 133.4 (×2), 133.2 (×4), 130.1 (×4), 130.0 (×4), 129.9 (×4), 129.8 (×4), 129.5 (×4), 129.0 (×4), 128.6 (×8), 128.5 (×4), 128.4 (×4), 115.5, 98.5, 98.4, 80.6, 71.5, 70.5 (×2), 70.4 (×2), 70.3 (×2), 69.4 (×2), 66.8 (×2), 62.9 (×4), 41.3, 34.4, 31.5, 22.7 (×2). HRMS (ESI-QqTOF) *m*/*z*: Calc. for C_79_H_74_NO_20_ [M+NH_4_]^+^ 1357.48323, found 1357.48118.

*Compound **10**:* The alkyne **8** (450 mg, 0.34 mmol) and the azidomethyl BODIPY derivative **9** [39] (99 mg, 0.3 mmol) were dissolved in THF (15 mL). Cu(I)-thiophene-2-carboxylate (57 mg, 0.3 mmol) was added, and the resulting solution was stirred at r.t. for 2 h. The crude was concentrated and purified by flash chromatography (Hexane:EtOAc, 6:4) to give **10** (344 mg, 70%). ^1^H-NMR (500 MHz; CDCl_3_): 8.04–7.68 (m, 20H), 7.51–7.08 (m, 28H), 6.59 (d, *J* = 4.2 Hz, 1H), 6.49 (d, *J* = 4.1 Hz, 1H), 6.36 (dd, *J* = 4.3, 1.9 Hz, 1H), 6.33 (dd, *J* = 4.3, 1.9 Hz, 1H), 6.09 (t, *J* = 10.0 Hz, 2H), 5.85–5.71 (m, 3H), 5.62–5.58 (m, 2H), 5.46 (t, *J* = 15.4 Hz, 1H), 5.38 (d, *J* = 15.4 Hz, 1H), 5.02 (d, *J* = 2.0 Hz, 1H), 5.01 (d, *J* = 1.9 Hz, 1H), 4.96–4.88 (m, 2H), 4.71–4.65 (m, 2H), 4.56–4.43 (m, 4H), 3.77 (t, *J* = 10.2 Hz, 2H), 3.33 (t, *J* = 9.1 Hz, 2H), 2.79 (d, *J* = 14.7 Hz, 1H), 2.73 (d, *J* = 14.9 Hz, 1H), 2.04 (m, 2H), 1.44 (m, 4H). ^13^C-NMR (125 MHz; CDCl_3_): 166.2 (×2), 165.6 (×4), 165.4 (×2), 145.4 (×2), 145.2 (×2), 144.0 (×2), 143.3 (×2), 138.7, 135.3, 134.4, 133.4 (×2), 133.3 (×2), 133.2 (×2), 133.1 (×2), 133.0, 132.3, 131.0, 130.9, 130.5, 130.3, 130.1, 130.0 (×2), 129.9 (×6), 129.8 (×6), 129.5, 129.4, 129.3, 129.2 (×2), 129.1 (×2), 128.6 (×2), 128.5 (×3), 128.4 (×2), 128.3 (×2), 128.1, 124.5, 119.2 (×2), 115.3, 98.5 (×2), 71.2, 70.8, 70.5 (×2), 70.4 (×2), 69.4 (×2), 66.7 (×2), 62.9 (×4), 51.6, 41.6, 34.4, 31.9, 28.4, 22.7. HRMS (ESI-QqTOF) *m*/*z*: Calc. for C_95_H_85_BF_2_N_5_O_20_ 1663.58799 [M+H]^+^, found 1663.58625.

#### 3.1.2. General Procedure for the Cross-Metathesis (CM) Reaction

The appropriate olefins were dissolved in dry CH_2_Cl_2_. Argon was bubbled through the solution for 10 min, and then the 2nd generation Hoveyda-Grubbs catalyst (5% mol) was added. The reaction mixture was refluxed for 12 h, and after which the air was bubbled through the solution. The solvent was evaporated in vacuo, and the residue was filtered through a FLORISIL pad and then purified by flash silica gel column chromatography.

*Compound **12**:* Olefin **10** (25 mg, 0.015 mmol) and Boc-protected O-allyl-l-tyrosine (**11**) (19.3 mg, 0.06 mmol), dissolved in CH_2_Cl_2_ (2 mL), were reacted according to the general method for the CM reaction. The residue was purified by flash silica gel column chromatography (CH_2_Cl_2_:MeOH 9:1) to give **12** (20 mg, 68%). ^1^H-NMR (500 MHz; CDCl_3_): 8.11–7.77 (m, 20H), 7.59–7.14 (m, 26H), 7.02 (d, *J* = 8.6 Hz, 2H), 6.79 (d, *J* = 8.7 Hz, 2H), 6.63 (m, 1H), 6.54 (m, 1H), 6.44 (m, 1H), 6.37 (m, 1H), 6.16 (t, *J* = 9.9 Hz, 2H), 5.85–5.50 (m, 8H), 5.08 (m, 2H), 4.76 (m, 2H), 4.57–4.47 (m, 8H), 3.85 (m, 2H), 3.44 (m, 2H), 3.06 (m, 2H), 2.83 (d, *J* = 15.4 Hz, 1H), 2.79 (d, *J* = 15.1 Hz, 1H), 2.17 (m, 2H), 1.51–1.44 (m, 6H), 1.26 (s, 9H). ^13^C-NMR (125 MHz; CDCl_3_): 166.3, 166.2 (×2), 165.7 (×2), 165.6, 165.5, 165.4, 157.8, 155.5, 145.5 (×2), 145.1 (×2), 143.9 (×2), 143.2, 135.3 (×2), 134.5, 134.3, 133.4 (×2), 133.3 (×2), 133.2 (×2), 133.1 (×2), 132.5 (×2), 131.1, 130.9, 130.5, 130.4, 130.3, 130.1, 130.0 (×2), 129.9 (×4), 129.8 (×4), 129.5, 129.4, 129.3, 129.2, 129.1, 129.0, 128.6 (×3), 128.5 (×2), 128.4, 128.3, 128.1, 126.3, 124.3, 119.4 (×2), 119.2 (×2), 115.3 (×2), 98.5 (×2), 71.5, 71.1, 70.5 (×6), 69.5, 69.4 (×2), 68.8 (×2), 66.6 (×2), 62.8 (×2), 54.4, 52.0, 41.5, 36.9, 33.0 (×2), 31.1, 29.8, 28.5 (×2), 22.9. HRMS (ESI-QqTOF) *m*/*z*: Calc. for C_110_H_102_BF_2_N_6_O_25_ [M+H]+ 1956.69925, found 1956.69249.; Calc. for C_110_H_101_BF_2_N_6_NaO_25_ [M+Na]+ 1978.68119, found 1978.70314.

*Compound **13**:* Olefin **10** (25 mg, 0.015 mmol), dissolved in CH_2_Cl_2_ (2 mL), was reacted according to the general method for the CM reaction. The residue was purified by flash silica gel column chromatography (Hexane:EtOAc, 1:1) to give **13** (11 mg, 44%). ^1^H-NMR (500 MHz; CDCl_3_): 8.11–7.74 (m, 38H), 7.56–7.08 (m, 56H), 6.65 (m, 2H), 6.55 (m, 2H), 6.43–6.39 (m, 4H), 6.18 (t, *J* = 10.1 Hz, 4H), 5.91–5.85 (m, 4H), 5.70 (bs, 4H), 5.52–5.42 (m, 6H), 5.11 (bs, 4H), 4.78–4.75 (m, 4H), 4.68–4.53 (m, 8H), 3.91–3.85 (m, 4H), 3.47–3.40 (m, 4H), 2.86–2.80 (m, 4H), 2.08–1.96 (m, 4H), 1.51–1.38 (m, 8H). ^13^C-NMR (125 MHz; CDCl_3_): 166.1 (×4), 165.5 (×4), 165.4 (×4), 165.3 (×4), 145.3 (×4), 145.2 (×4), 143.9 (×4), 143.2 (×4), 135.3 (×4), 135.2 (×4), 134.5 (×4), 134.4 (×4), 133.4 (×4), 133.3 (×4), 133.2 (×4), 133.1 (×4), 132.3 (×2), 131.1 (×2), 131.0 (×2), 130.5 (×4), 130.3 (×4), 130.2 (×4), 130.1 (×6), 130.0 (×6), 129.9 (×8), 129.8 (×4), 129.7 (×4), 129.5 (×4), 129.3 (×4), 129.2 (×4), 129.1 (×4), 129.0 (×4), 128.6 (×4), 128.5 (×4), 128.4 (×3), 128.3 (×3), 128.1 (×2), 119.1 (×4), 98.5 (×4), 71.4 (×2), 71.2 (×2), 70.6 (×8), 69.4 (×4), 66.6 (×4), 62.9 (×4), 51.6 (×2), 33.6 (×2), 29.9 (×4), 28.2 (×2). ^19^F-NMR (376 MHz; CDCl_3_): –144.7 (dq, *J*_F,F_ = 103 Hz, *J*_B-F_ = 27.1 Hz), –146.7 (dq, *J*_F,F_ = 103 Hz, *J*_B-F_ = 28.0 Hz). ^11^B-NMR (128 MHz; CDCl_3_): 0.16 (t, *J*_B_-_F_ = 28.8 Hz). HRMS (ESI-QqTOF) *m*/*z*:Calc. for C_188_H_161_B_2_F_4_N_10_NaO_40_ [M+Na]+ 3319.08679, found 3319.10065. Unreacted olefin **10** (11 mg, 44%) could also be recovered.

*Compound **14**:* A solution of compound **13** (23 mg, 0.007 mmol) in MeOH–CH_2_Cl_2_ (V:V/2:1, 4 mL) solution was treated with NaOMe (24 mg, 0.45 mmol, 3 equiv/OBz). After stirring at room temperature for 12 h, the solution was neutralized with ion-exchange resin (H+), then filtered and concentrated. The residue was purified by column chromatography on silica gel (EtOAc/MeOH/H_2_O: 7/2/1) to give **14** (9.8 mg, 90%). ^1^H-NMR (500 MHz; CD_3_OD): 7.95 (s, 2H), 7.65–7.35 (m, 12H), 6.61–6.51 (m, 8H), 5.51 (m, 4H), 5.30–5.20 (m, 2H), 4.64 (s, 4H), 3.81–3.48 (m, 28H), 3.13–3.00 (m, 4H), 2.64–2.46 (m, 4H), 1.83 (m, 4H), 1.33–1.08 (m, 8H). ^13^C-NMR (125 MHz; CD_3_OD): 146.6 (×2), 145.0 (×2), 137.2 (×2), 137.1 (×2), 135.4 (×2), 134.5 (×2), 131.8 (×4), 131.6 (×4), 131.0 (×6), 129.5 (×4), 124.8 (×4), 119.9 (×4), 102.3 (×2), 102.2 (×2), 74.8 (×4), 72.7 (×4), 72.0 (×4), 71.0 (×2), 68.6 (×4), 62.7 (×4), 54.8 (×2), 42.3 (×2), 32.7 (×2), 30.7 (×2), 28.9 (×2), 23.7 (×2). ^19^F-NMR (376 MHz; CD_3_OD): No signals observed. ^11^B-NMR (128 MHz; CD_3_OD): 1.45 (bs). HRMS (ESI-QqTOF) *m*/*z*: Calc. for C_76_H_93_B_2_N_10_O_24_ [M+H]+ 155.65503, found 1551.66285.

### 3.2. Spectroscopy

The photophysical properties were registered in diluted solutions (around 2 × 10^−6^ M) prepared by adding the corresponding solvent (spectroscopic grade) to the residue from the adequate amount of a concentrated stock solution in acetone (compounds **10**, **12**, and **13**) or methanol (compound **14**). UV-Vis absorption and fluorescence spectra were recorded on a Varian model CARY 4E spectrophotometer and a SPEX Fluorolog 322 spectrofluorimeter, respectively, using quartz cuvettes with an optical pathway of 1 cm. Fluorescence quantum yield (ϕ) was obtained by using the commercial PM546 dye as reference (ϕ^r^ = 0.85 in ethanol). Radiative decay curves were registered with the time correlated single-photon counting technique (Edinburgh Instruments, model FL920), equipped with a microchannel plate detector (Hamamatsu C4878) of picosecond time-resolution (20 ps). Fluorescence emission was monitored at the maximum emission wavelength after excitation by means of a diode laser (PicoQuant, model LDH470) with 150 ps full width at half maximum (FWHM) pulses. The fluorescence lifetime (τ) was obtained after the deconvolution of the instrumental response signal from the recorded decay curves by means of an iterative method. The goodness of the exponential fit was controlled by statistical parameters (chi-square and the analysis of the residuals).

## Figures and Tables

**Table 1:** Photophysical properties of diluted solutions (2 μM) of boron-dipyrromethene (BODIPY)-labeled tetravalent (**10** and **12)** and hexavalent (**13** and **14**) derivatives in different solvents.

Dye	Solvent	λ_ab_ (nm)	ε_max_ (10^4^ M^−1^ cm^−1^)	λ_fl_ (nm)	ϕ	τ (ns)
**10**	Ethyl acetate	502.5	5.0	515.5	0.90	7.02
	Acetone	502.5	5.1	515.0	0.80	7.05
	Acetonitrile	502.0	5.0	515.0	0.82	7.53
**12**	Ethyl acetate	503.0	4.0	516.0	0.78	6.14
	Acetone	502.5	4.2	516.5	0.55	5.04
	Acetonitrile	502.5	3.5	515.5	0.60	5.20
**13**	Ethyl acetate	503.0	10.2	518.0	0.70	5.69
	Acetone	503.0	9.3	517.5	0.50	5.04
	Acetonitrile	502.5	9.5	516.0	0.55	5.14
**14**	Methanol	501.5	8.5	518.0	0.40	4.38
	Water	501.5	8.0	518.0	0.30	3.90

Absorption (λ_ab_) and fluorescence (λ_fl_) wavelengths, molar absorption at the maximum wavelength (ε_max_), fluorescence quantum yield (ϕ), and lifetime (τ).

## Supplementary Materials

Supplementary materials are available online. Figures S1–S26: Copies of 1D and 2D NMR spectra for all compounds.
