# Supplementary material for: A Malonyl-Based Scaffold for Conjugatable Multivalent Carbohydrate-BODIPY Presentations
Source: Molecules. 2019 May 29;24(11):2050. doi: 10.3390/molecules24112050 (PMC6600552; doi:10.3390/molecules24112050)

**Supplementary materials**

# A Malonyl-Based Scaffold for Conjugatable Multivalent Carbohydrate-BODIPY presentations

**Clara Uriel*[1], Rebeca Sola-Llano[2], Jorge Bañuelos[2], Ana M. Gomez [1], J. Cristobal Lopez*[1]**

[1] Instituto de Química Orgánica General, Consejo Superior de Investigaciones Científicas (*IQOG-CSIC*), Juan de la Cierva 3, 28006 Madrid, Spain. clara.uriel@csic.es (C.U.); jc.lopez@csic.es (J.C.L.) ana.gomez@csic.es (A.M.G.)

[2] Departamento Química Física, Universidad del País Vasco (*UPV/EHU*), Aptdo 644, 48080, Bilbao, Spain; rebeca.sola@ehu.es (R.S.L.); jorge.banuelos@ehu.es (J.B.)

* Correspondence: clara.uriel@csic.es (C.U.); jc.lopez@csic.es (J.C.L.) (phone +34912587616)

General methods ........ 2
Figure S1. $^{1}$H-NMR spectrum of Dimethyl 2-(pent-4'-enyl) malonate ........ 3
Figure S2. $^{1}$H-NMR spectrum of compound **5** ........ 3
Figure S3. $^{13}$C-NMR spectrum of compound **5** ........ 4
Figure S4. HSQC-NMR spectrum of compound **5** ........ 4
Figure S5. $^{1}$H-NMR spectrum of compound **6** ........ 5
Figure S6. $^{13}$C-NMR spectrum of compound **6** ........ 5
Figure S7. HSQC-NMR spectrum of compound **6** ........ 6
Figure S8. $^{1}$H-NMR spectrum of compound **8** ........ 6
Figure S9. $^{13}$C-NMR spectrum of compound **8** ........ 7
Figure S10. HSQC-NMR spectrum of compound **8** ........ 7
Figure S11. $^{1}$H-NMR spectrum of compound **10** ........ 8
Figure S12. $^{13}$C-NMR spectrum of compound **10** ........ 8
Figure S13. HSQC-NMR spectrum of compound **10** ........ 9
Figure S14. $^{1}$H-NMR spectrum of compound **12** ........ 9
Figure S15. $^{13}$C-NMR spectrum of compound **12** ........ 10
Figure S16. HSQC-NMR spectrum of compound **12** ........ 10
Figure S17. $^{1}$H-NMR spectrum of compound **13** ........ 11
Figure S18. $^{13}$C-NMR spectrum of compound **13** ........ 11
Figure S19. HSQC-NMR spectrum of compound **13** ........ 12
Figure S20. $^{11}$B-NMR spectrum of compound **13** ........ 12
Figure S21. $^{19}$F-NMR spectrum of compound **13** ........ 13
Figure S22. $^{1}$H-NMR spectrum of compound **14** ........ 13
Figure S23. $^{13}$C-NMR spectrum of compound **14** ........ 14
Figure S24. HSQC-NMR spectrum of compound **14** ........ 14
Figure S25. $^{11}$B-NMR spectrum of compound **14** ........ 15
Figure S26. $^{19}$F-NMR spectrum of compound **14** ........ 15

## General methods

General Information. All solvents and reagents were obtained commercially and used as received unless stated otherwise. Residual water was removed from starting compounds by repeated coevaporation. Reactions were executed at ambient temperatures unless stated otherwise. All moisture-sensitive reactions were performed in dry flasks fitted with glass stoppers or rubber septa under a positive pressure of argon. Air- and moisture-sensitive liquids and solutions were transferred by syringe or stainless steel cannula.. Anhydrous $MgSO_4$ or $Na_2SO_4$ were used to dry organic solutions during workup, and evaporation of the solvents was performed under reduced pressure using a rotary evaporator. Flash column chromatography was performed using 230–400 mesh silica gel. Thin-layer chromatography was conducted on Kieselgel 60 F254. Spots were observed first under UV irradiation (254 nm) then by charring with a solution of 20% aqueous $H_2SO_4$ (200 mL) in AcOH (800 mL). $^1H$ and $^{13}C$ NMR spectra were recorded in $CDCl_3$ at 300, 400, or 500 MHz and 75, 101, or 126 MHz, respectively. Chemical shifts are expressed in parts per million (δ scale) downfield from tetramethylsilane and are referenced to residual protium in the NMR solvent ($CHCl_3$: δ 7.25 ppm). Coupling constants (J) are given in Hz. All presented $^{13}C$ NMR spectra are proton-decoupled. Mass spectra were recorded by direct injection with a Accurate Mass Q-TOF LC/MS spectrometer equipped with an electrospray ion source in positive mode.

Figure S1. $^1$H-NMR spectrum of Dimethyl 2-(pent-4'-enyl) malonate

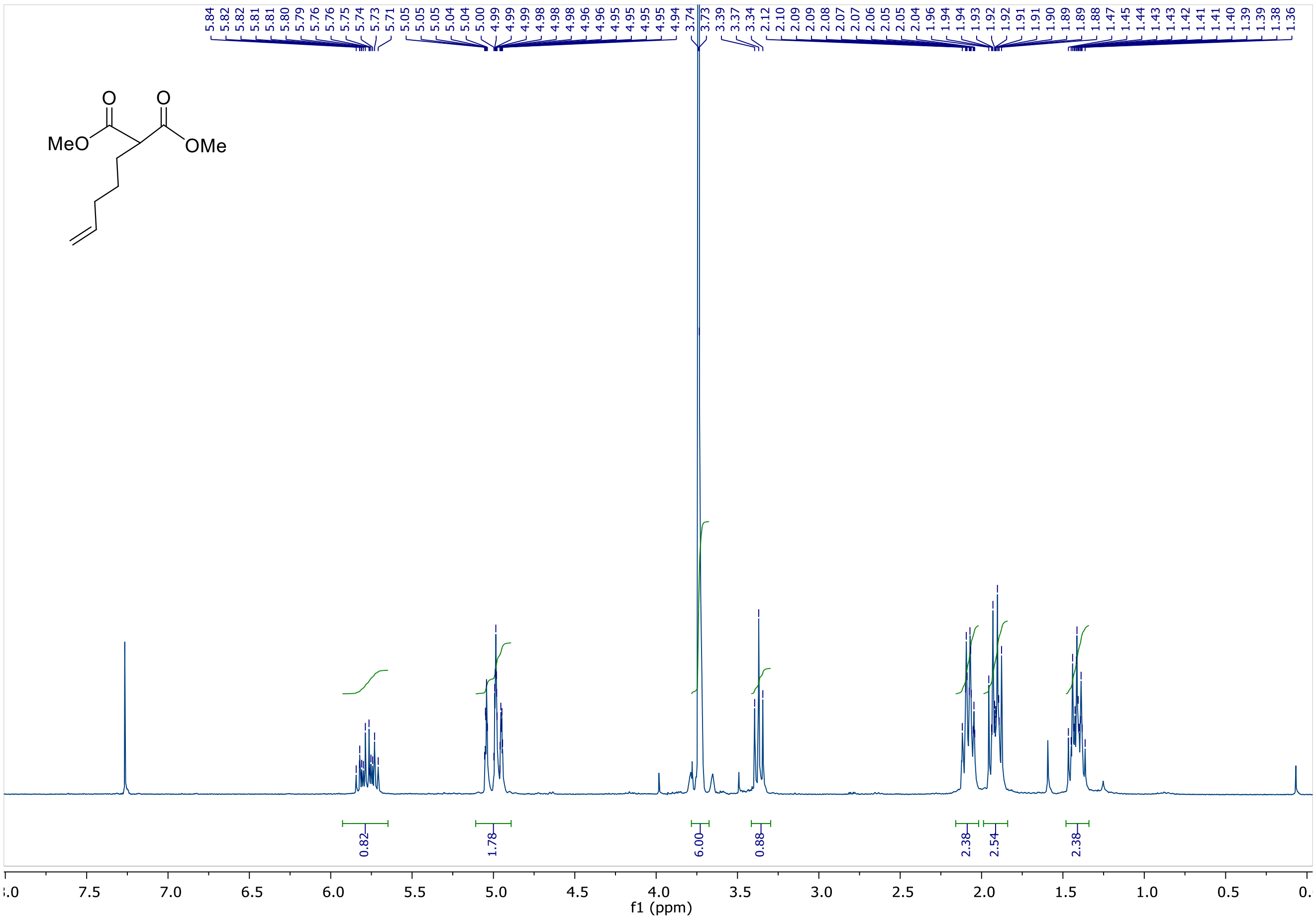

Figure S2. $^1$H-NMR spectrum of compound **5**

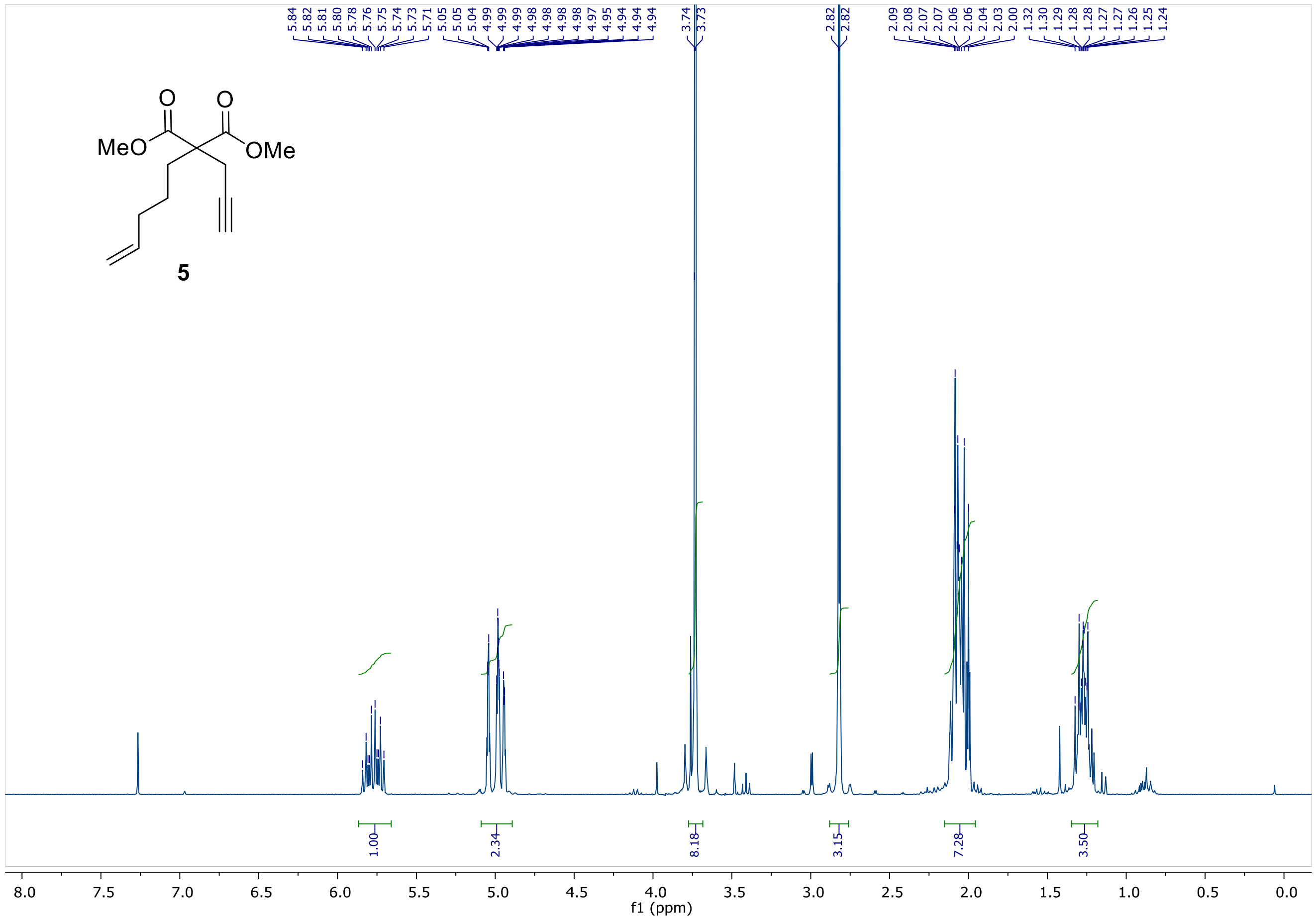

Figure S3. $^{13}$C-NMR spectrum of compound **5**

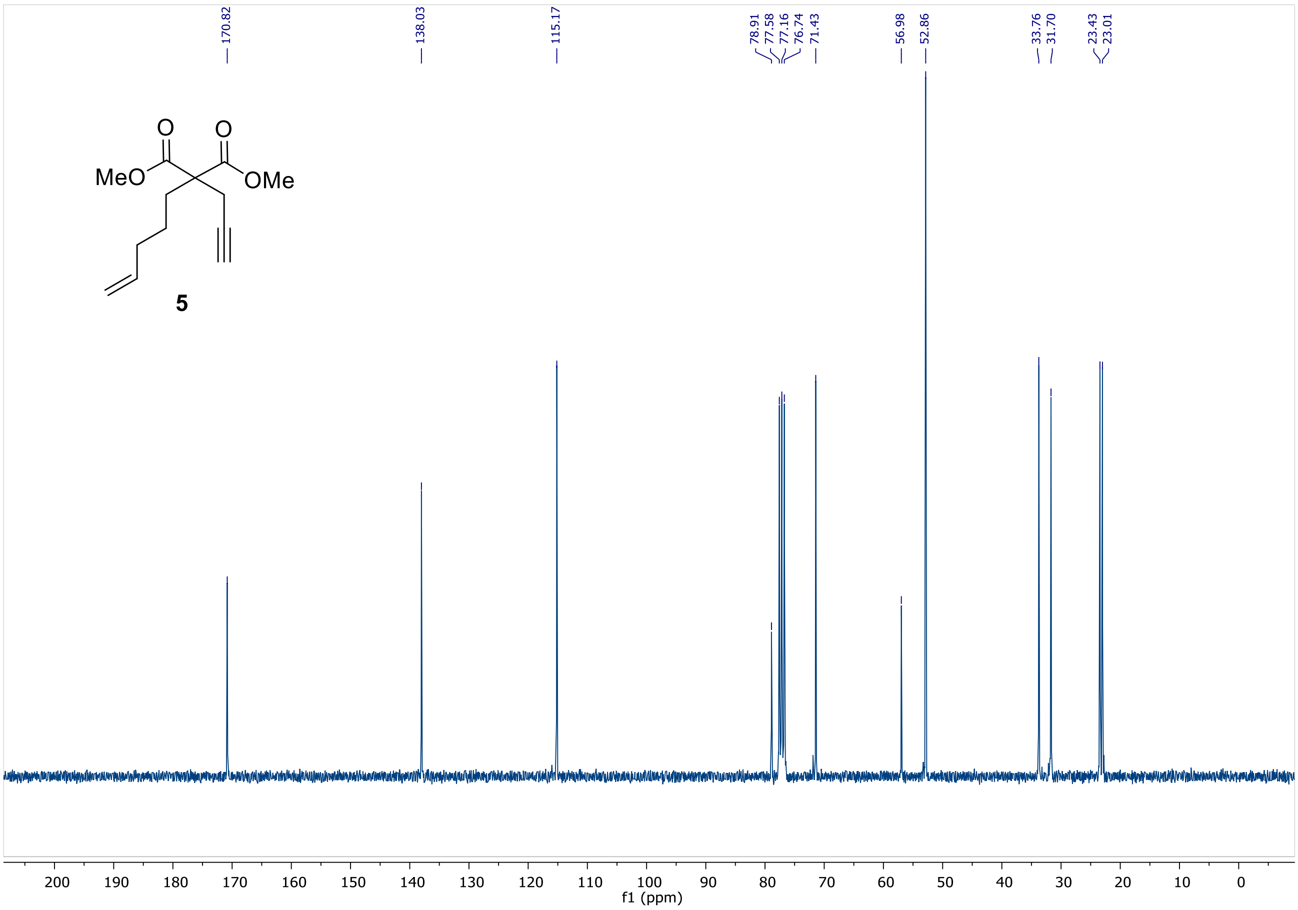


Figure S4. HSQC-NMR spectrum of compound **5**

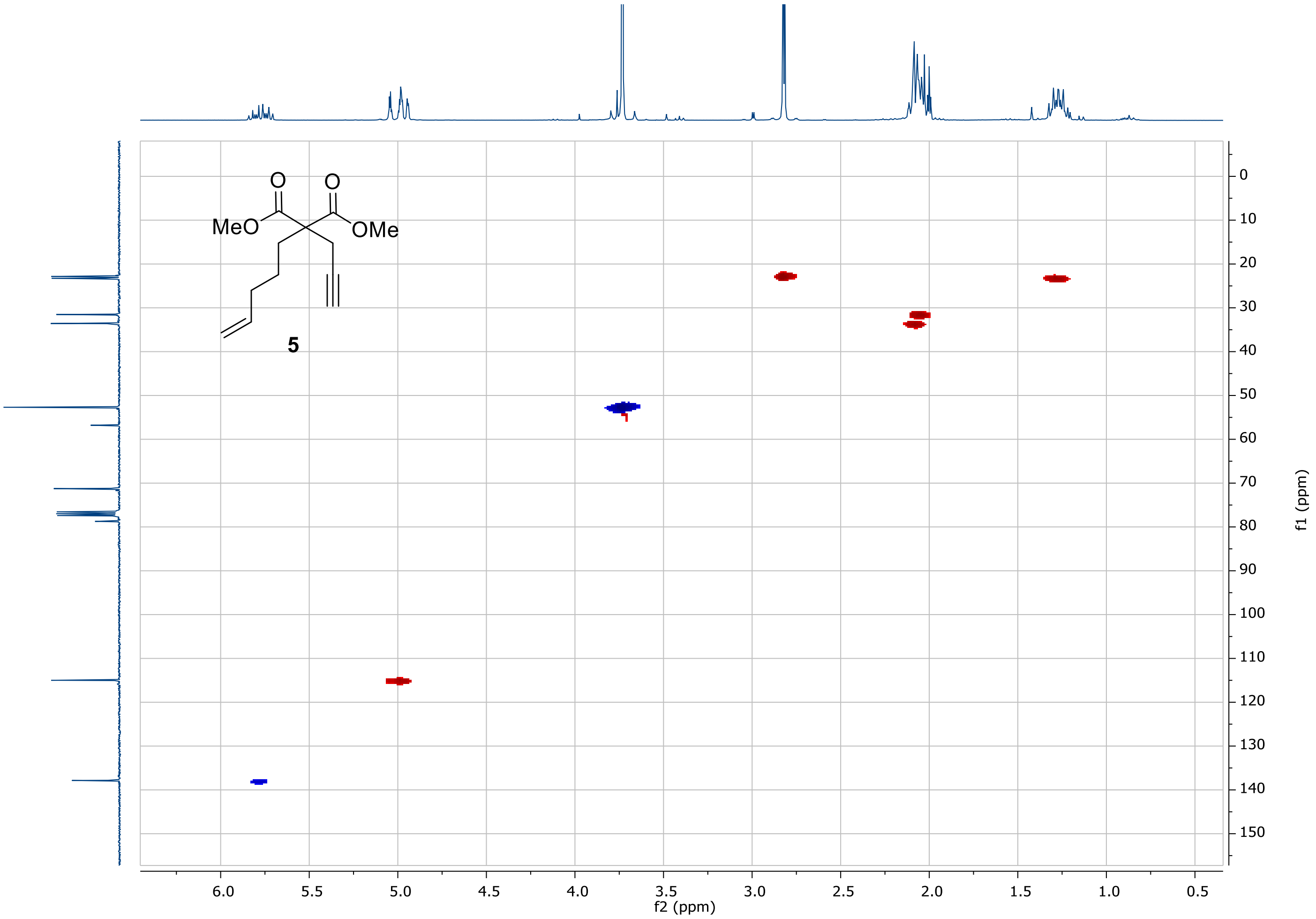

Figure S5. $^{1}$H-NMR spectrum of compound **6**

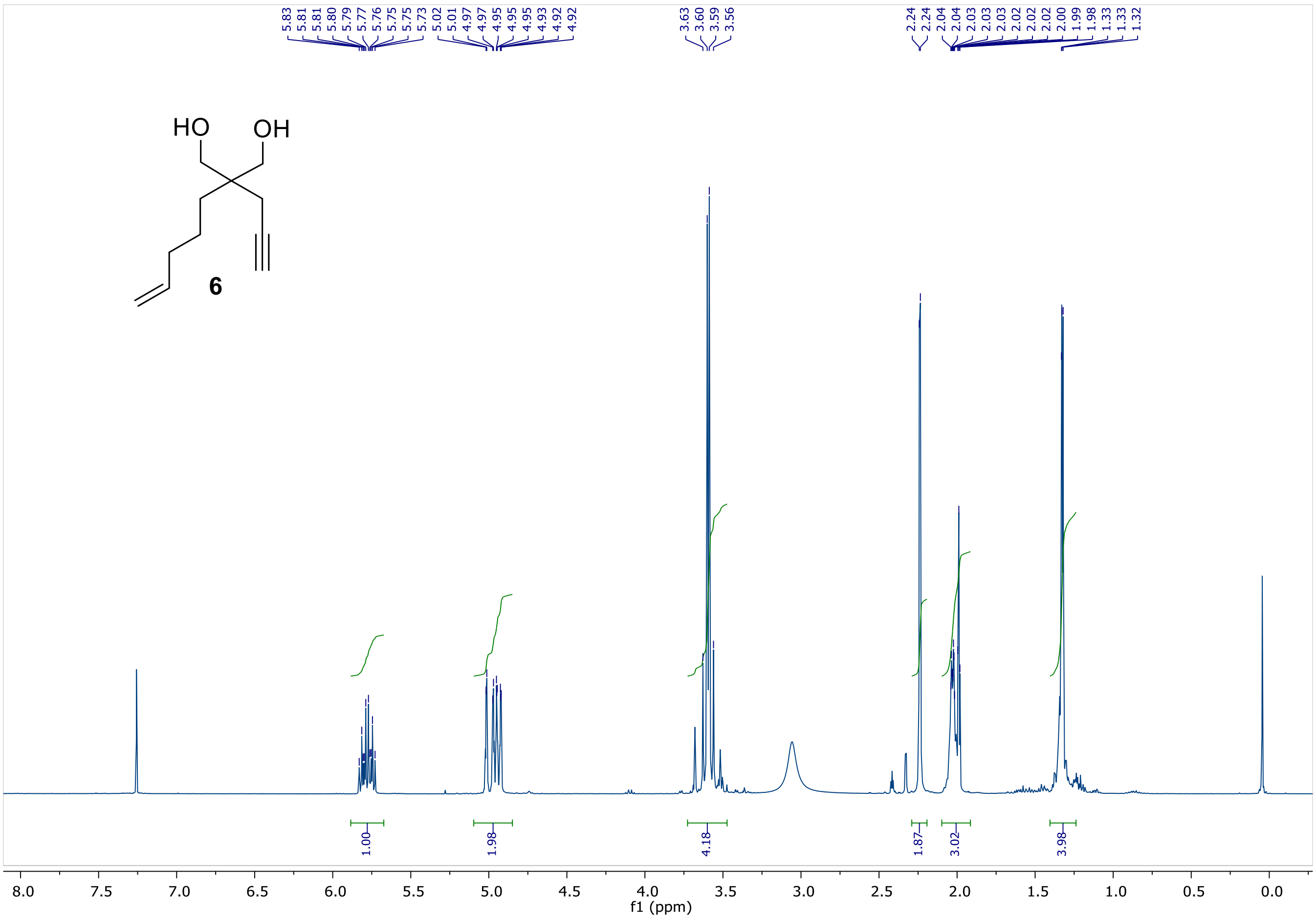


Figure S6. $^{13}$C-NMR spectrum of compound **6**

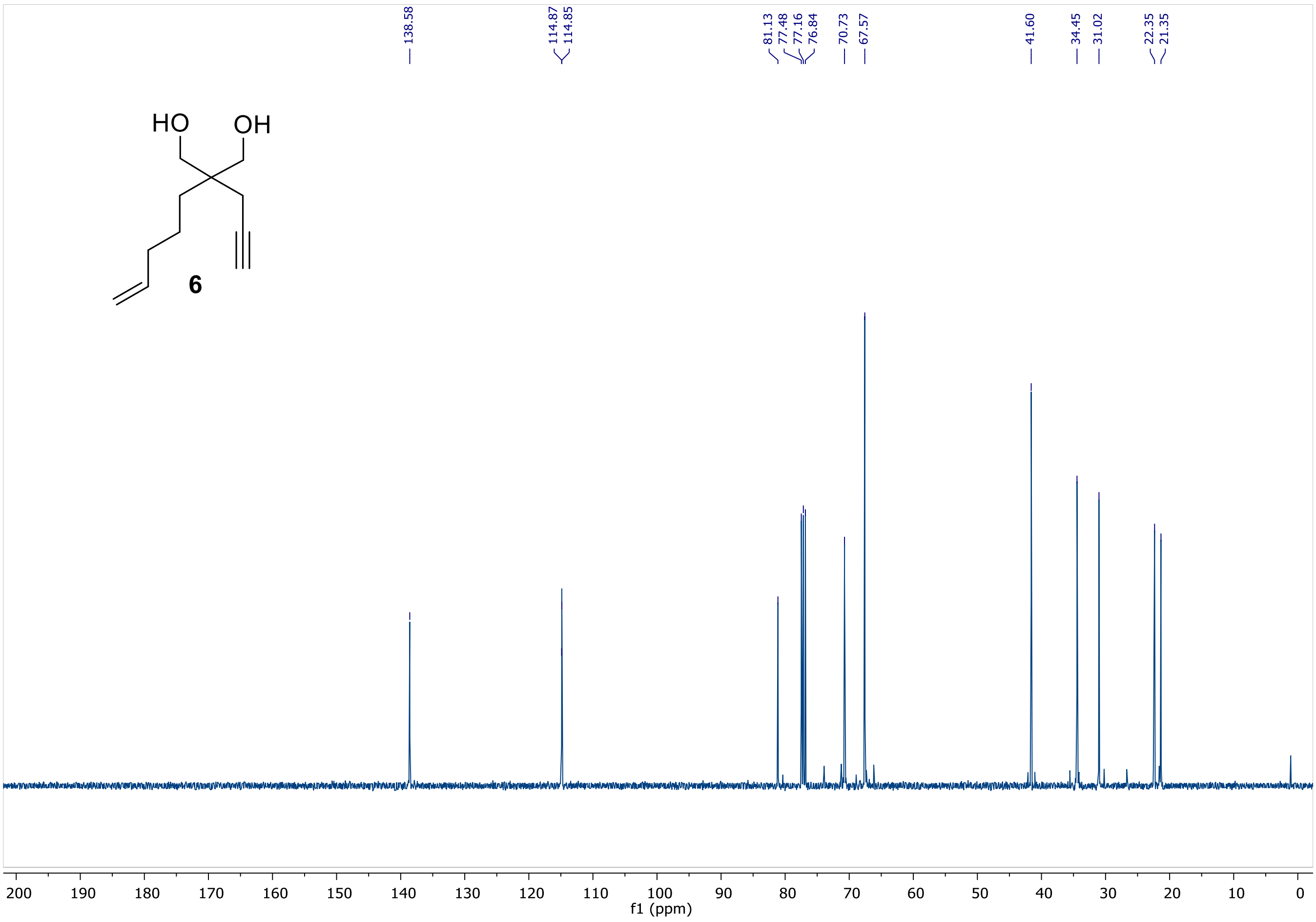

Figure S7. HSQC-NMR spectrum of compound **6**

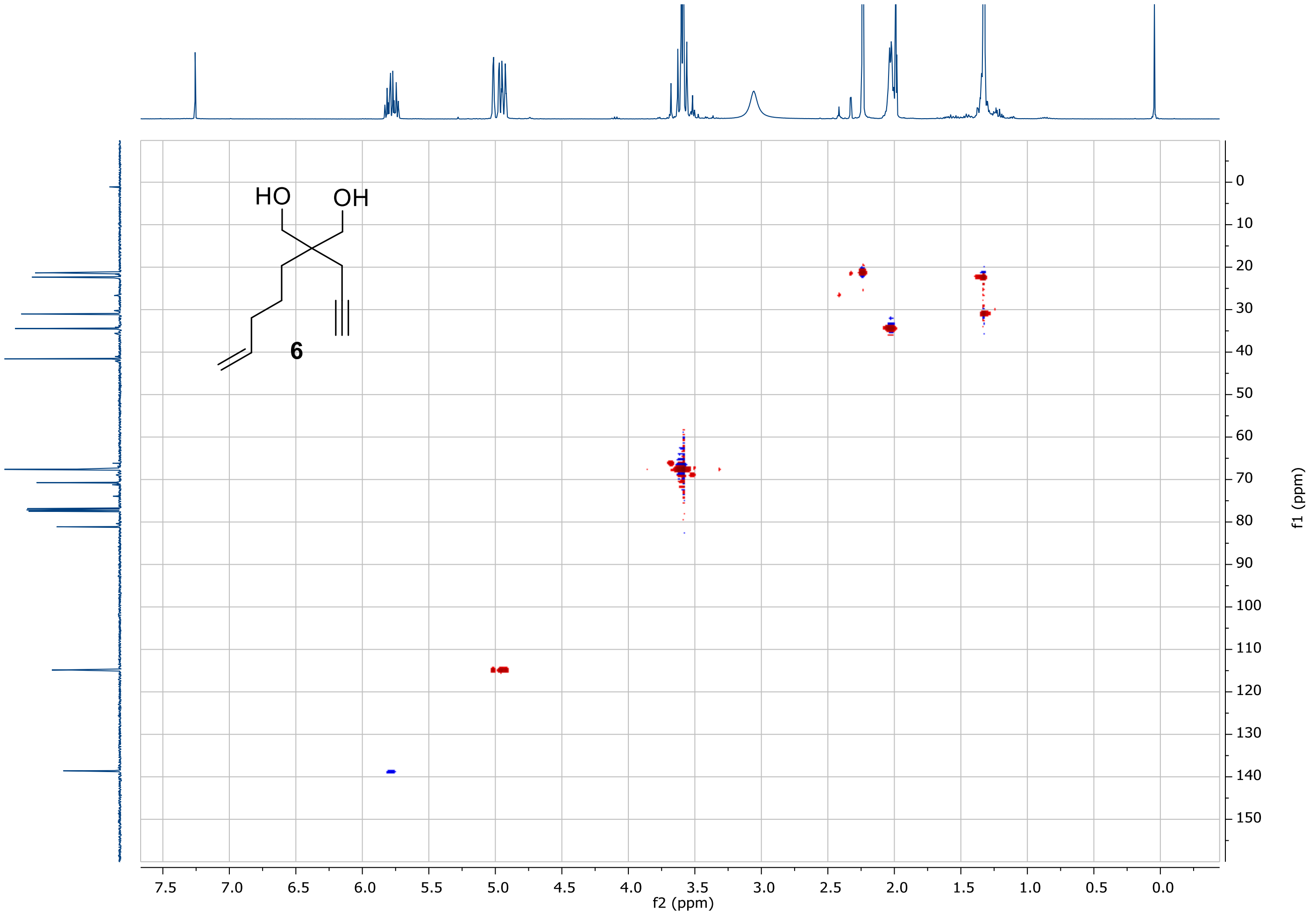

Figure S8. $^{1}$H-NMR spectrum of compound **8**

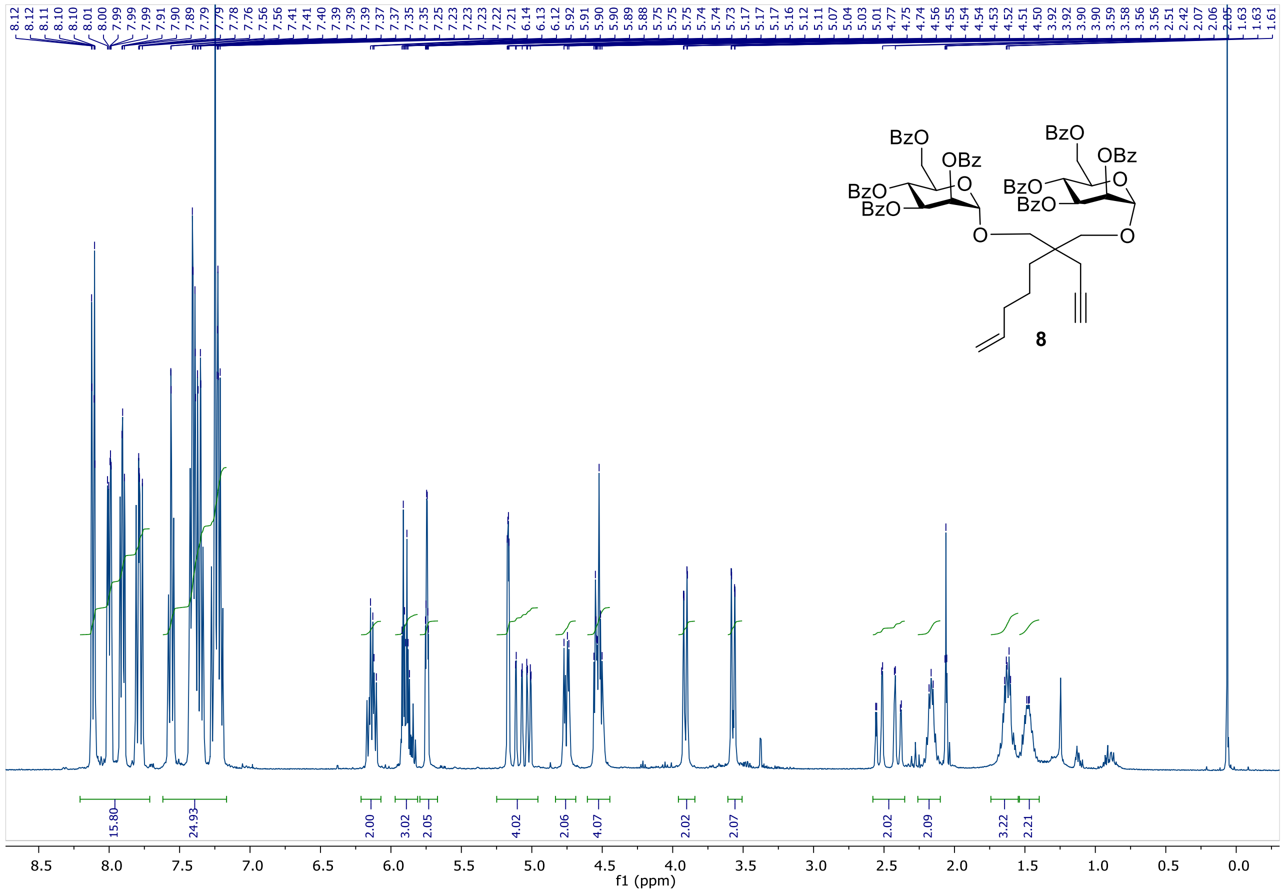

Figure S9. $^{13}$C-NMR spectrum of compound **8**

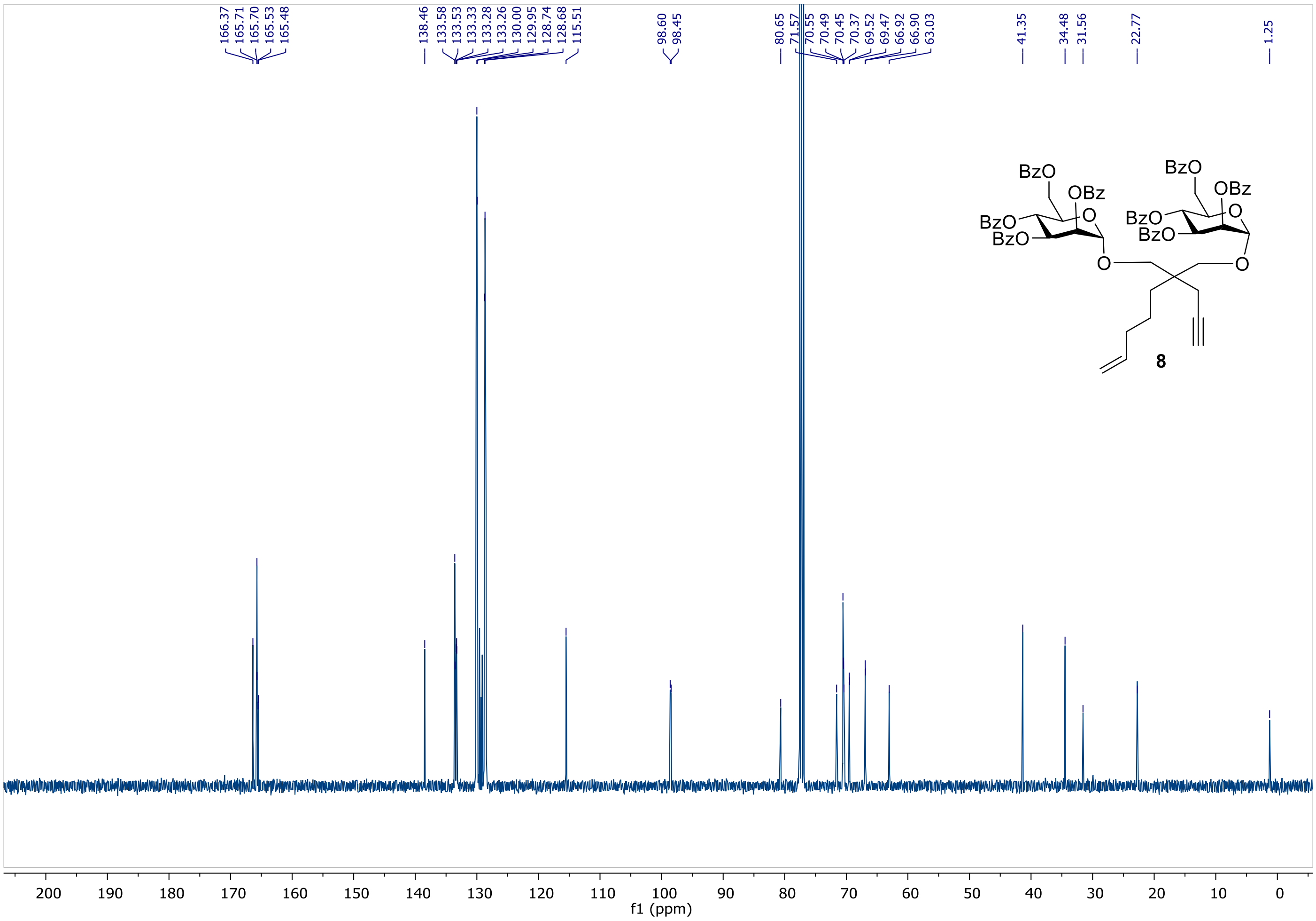


Figure S10. HSQC-NMR spectrum of compound **8**

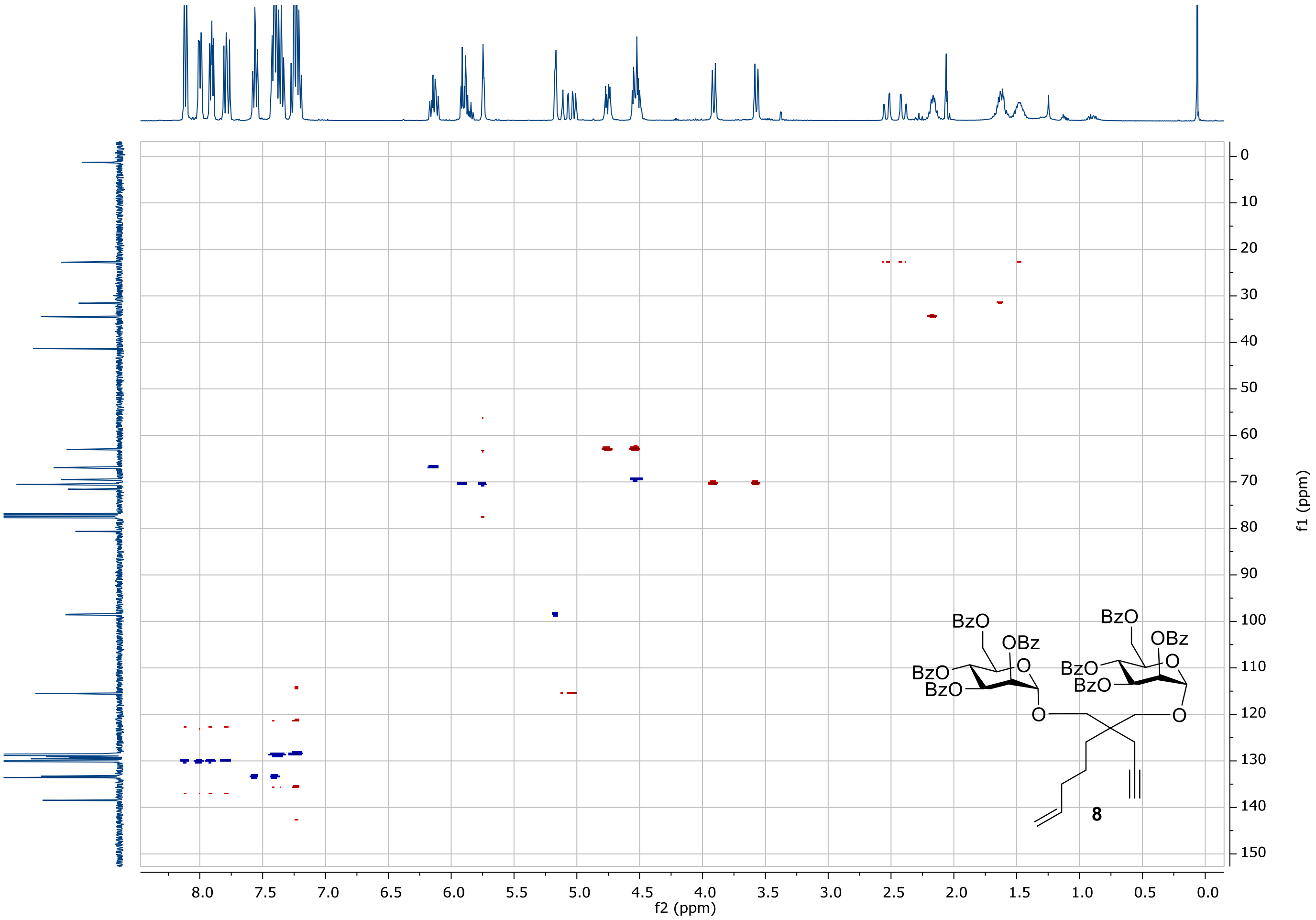

Figure S11. $^{1}$H-NMR spectrum of compound **10**

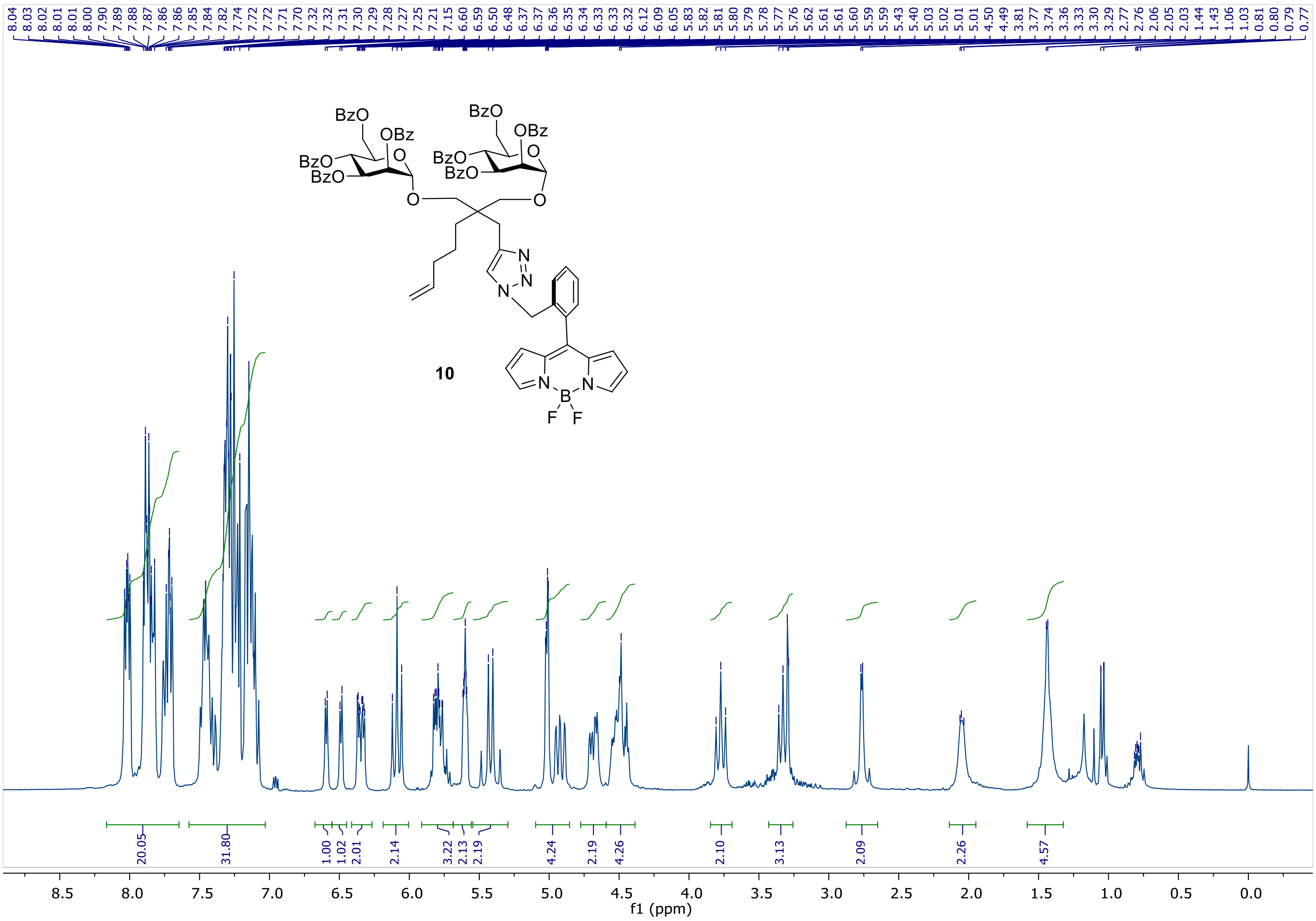


Figure S12. $^{13}$C-NMR spectrum of compound **10**

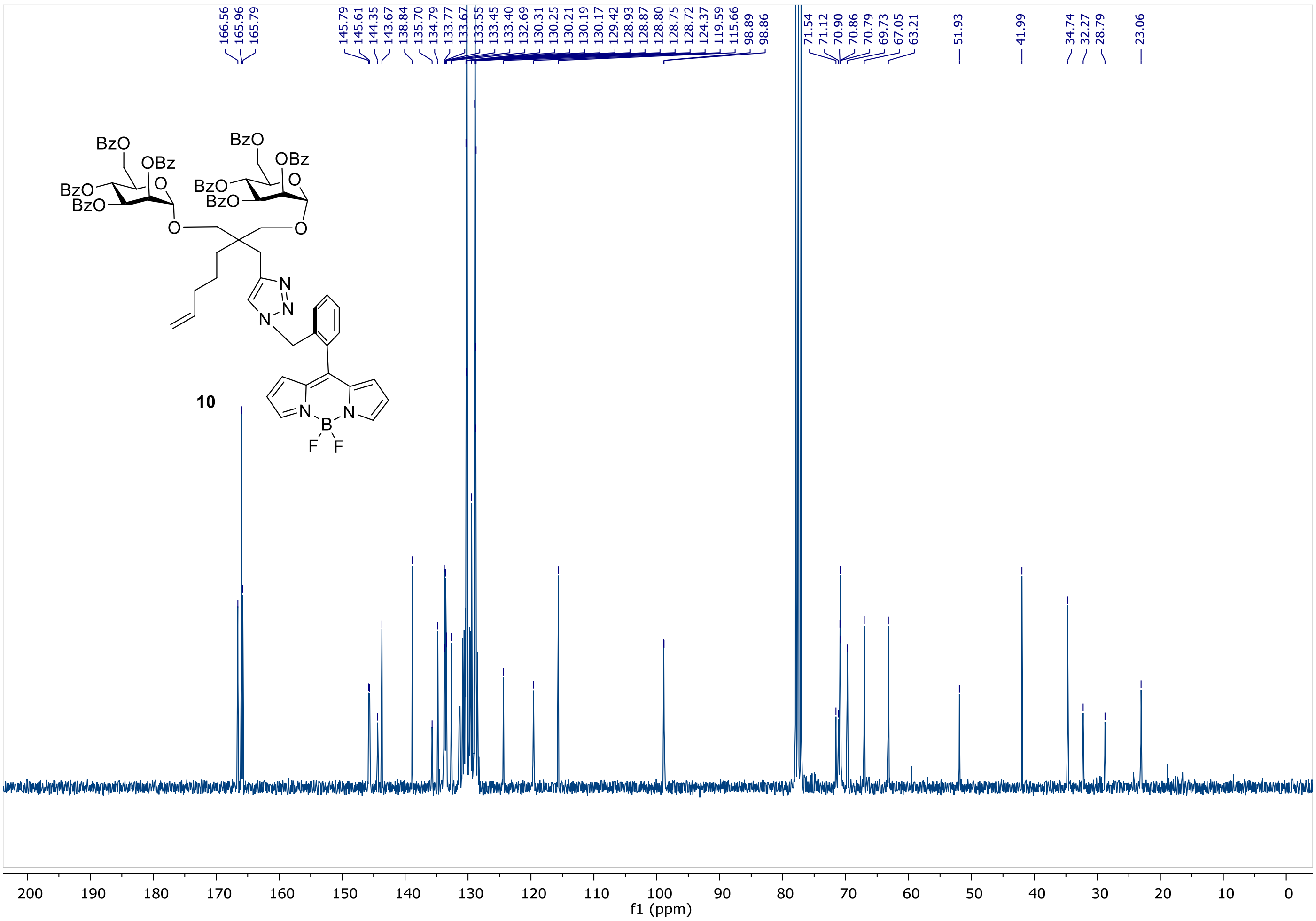

Figure S13. HSQC-NMR spectrum of compound **10**

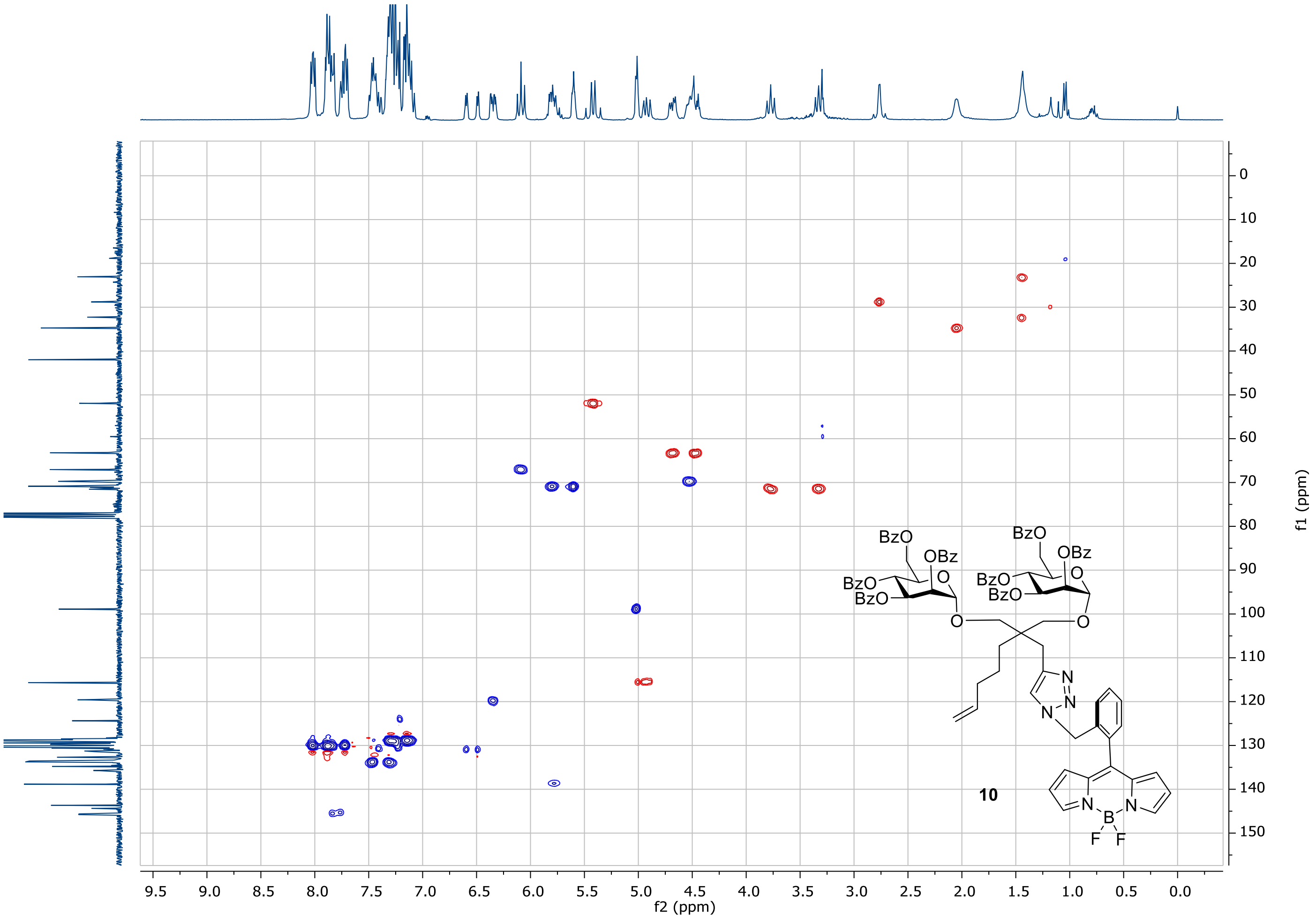


Figure S14. $^{1}$H-NMR spectrum of compound **12**

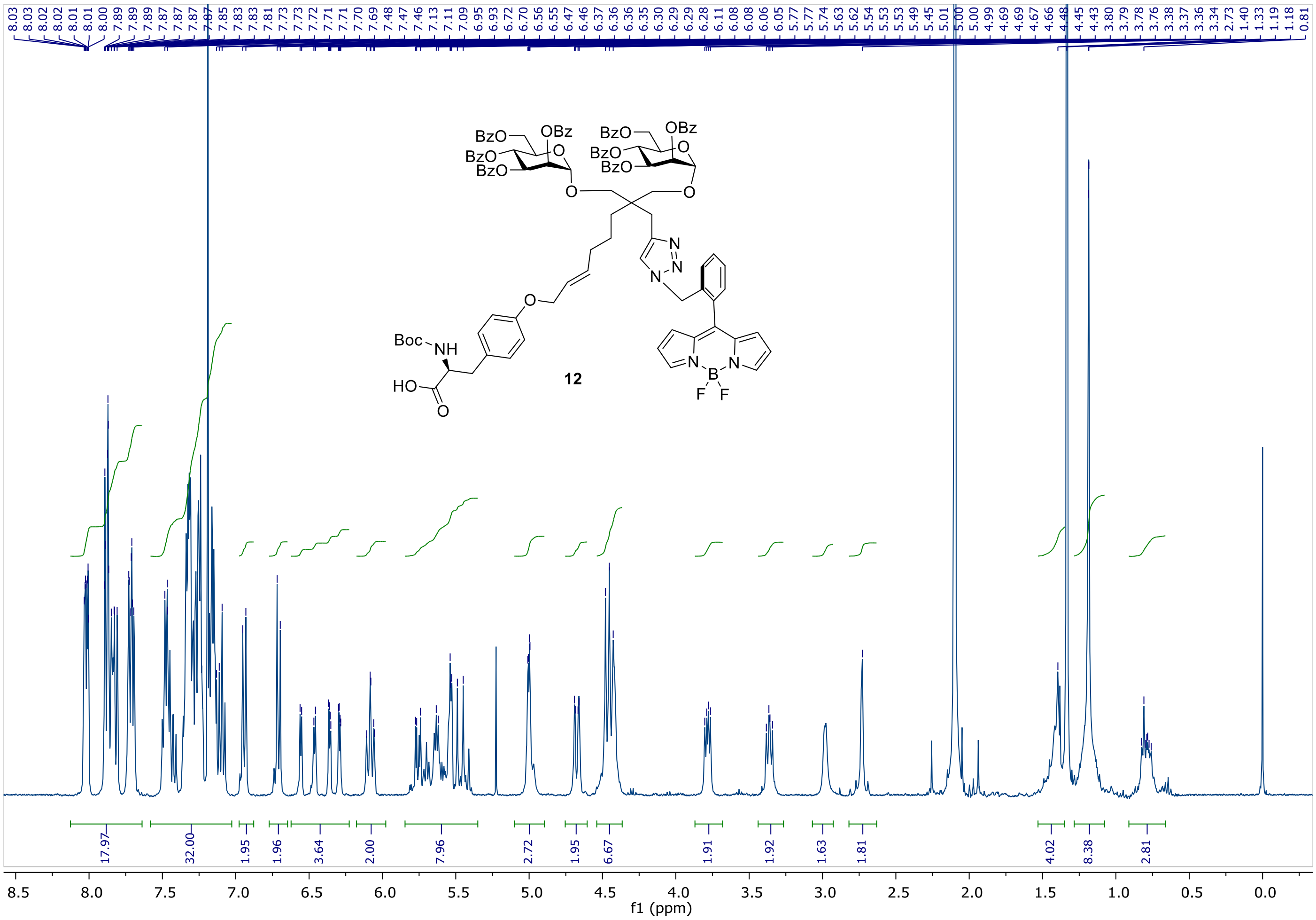

Figure S15. $^{13}C$-NMR spectrum of compound **12**

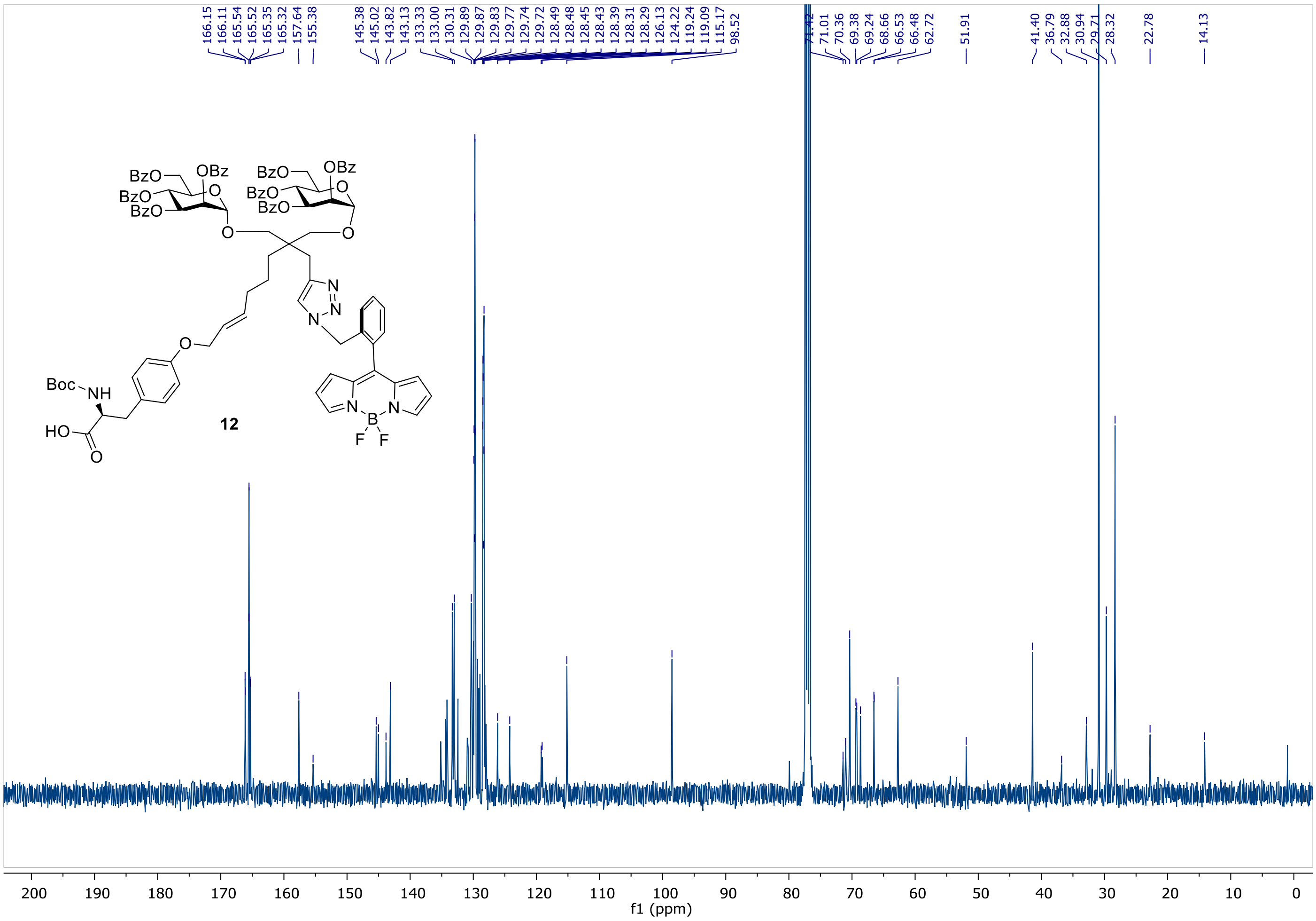


Figure S16. HSQC-NMR spectrum of compound **12**

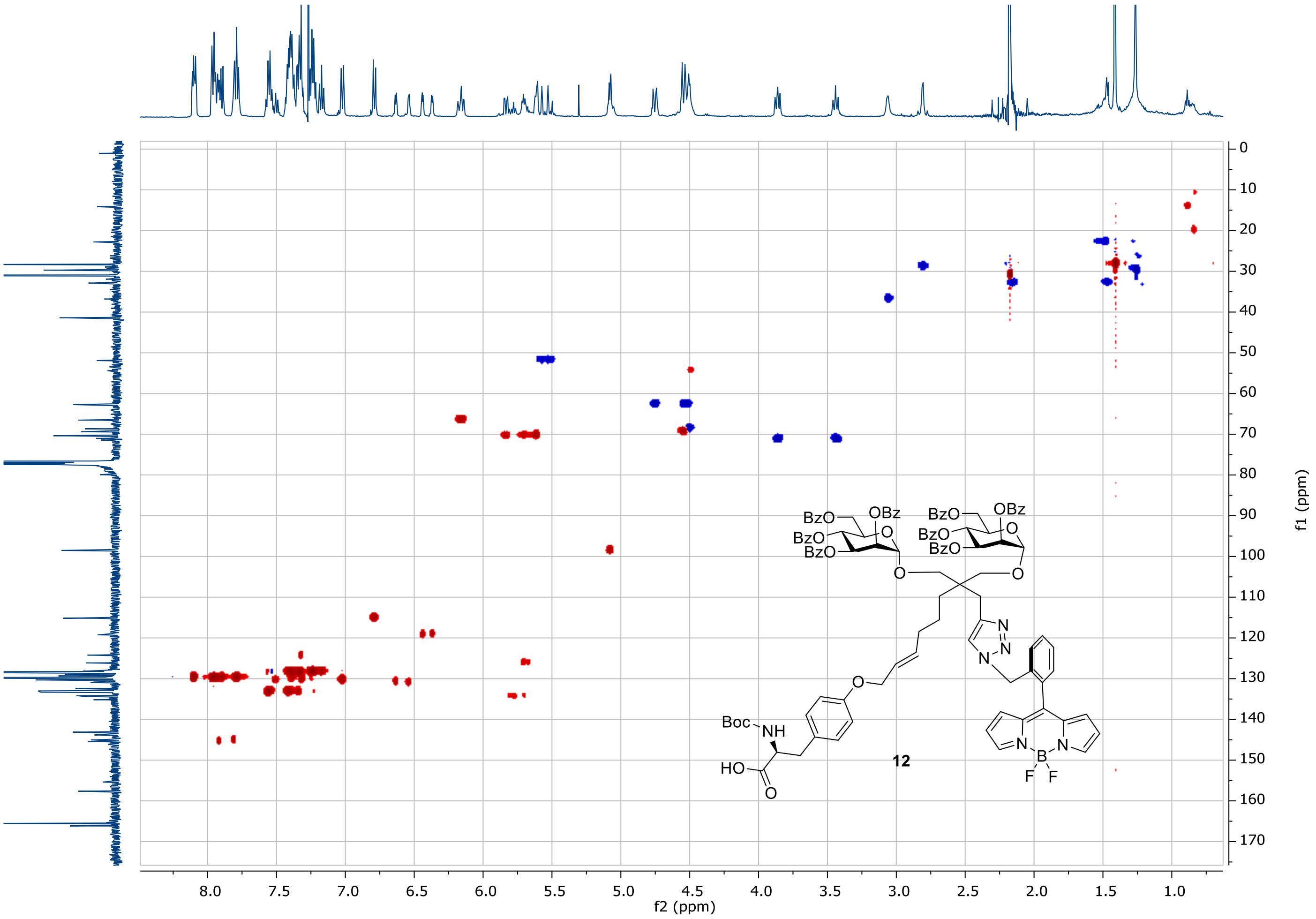

Figure S17. $^{1}$H-NMR spectrum of compound **13**

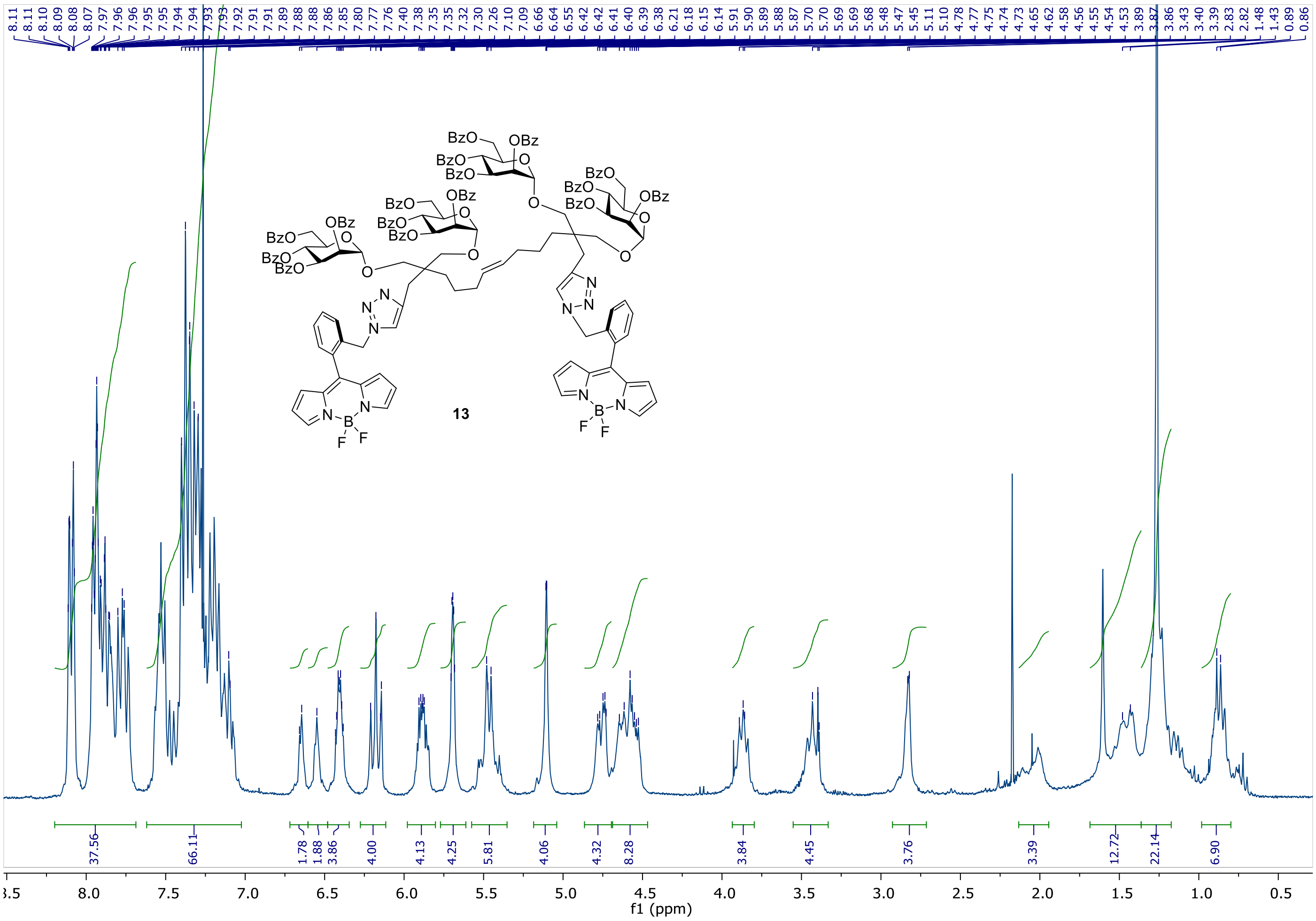

Figure S18. $^{13}$C-NMR spectrum of compound **13**

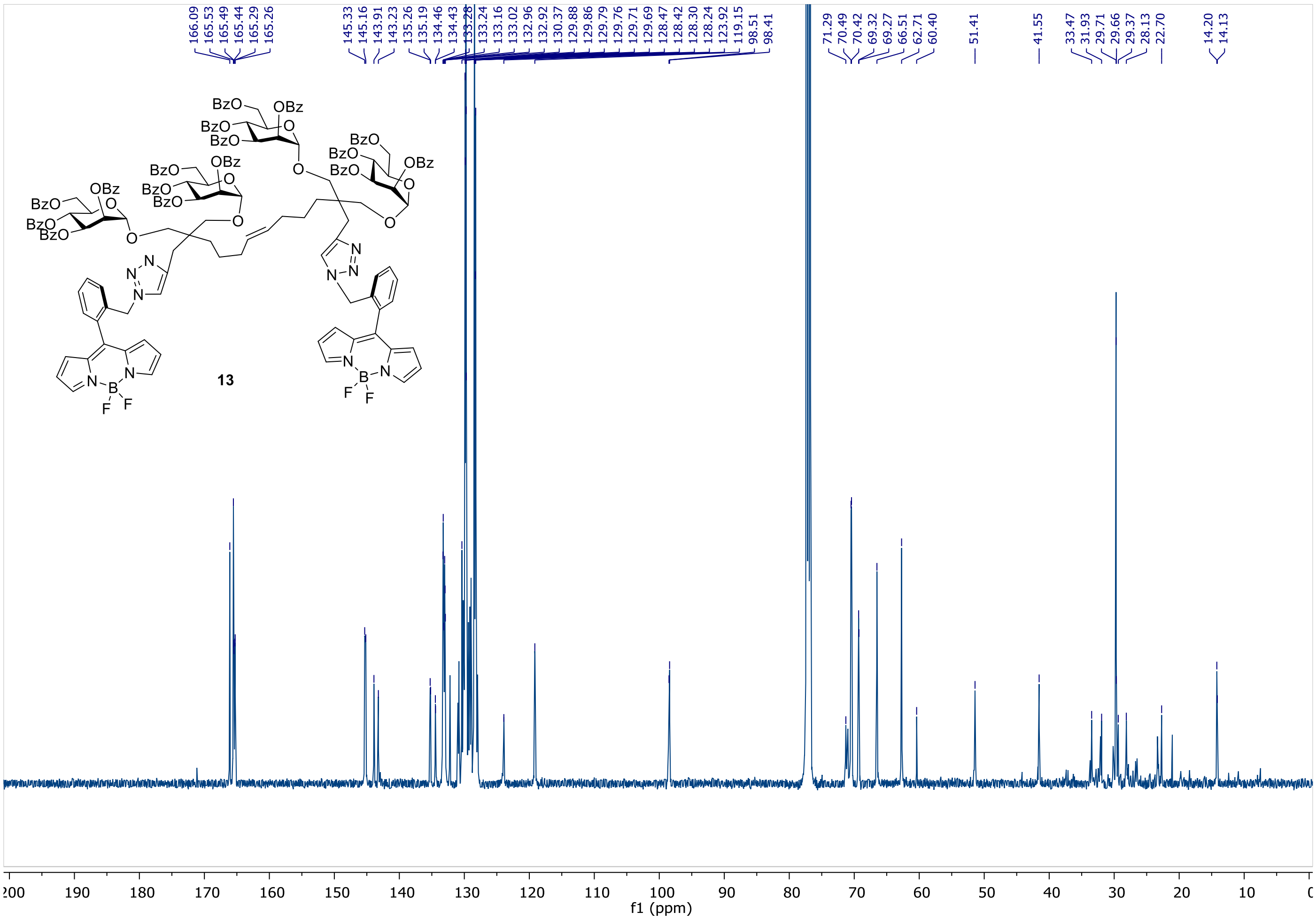

Figure S19. HSQC-NMR spectrum of compound **13**

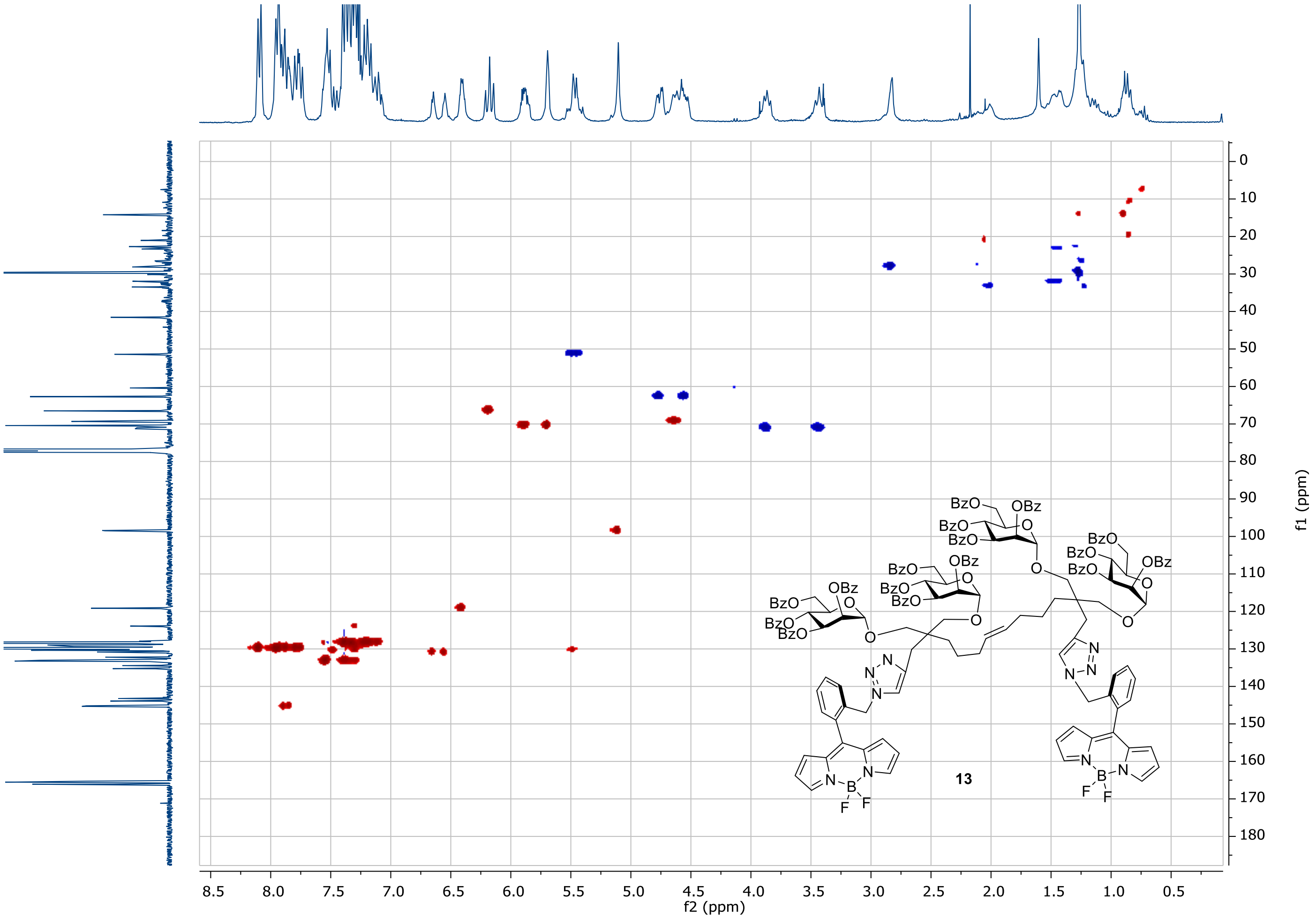


Figure S20. $^{11}$B-NMR spectrum of compound **13**

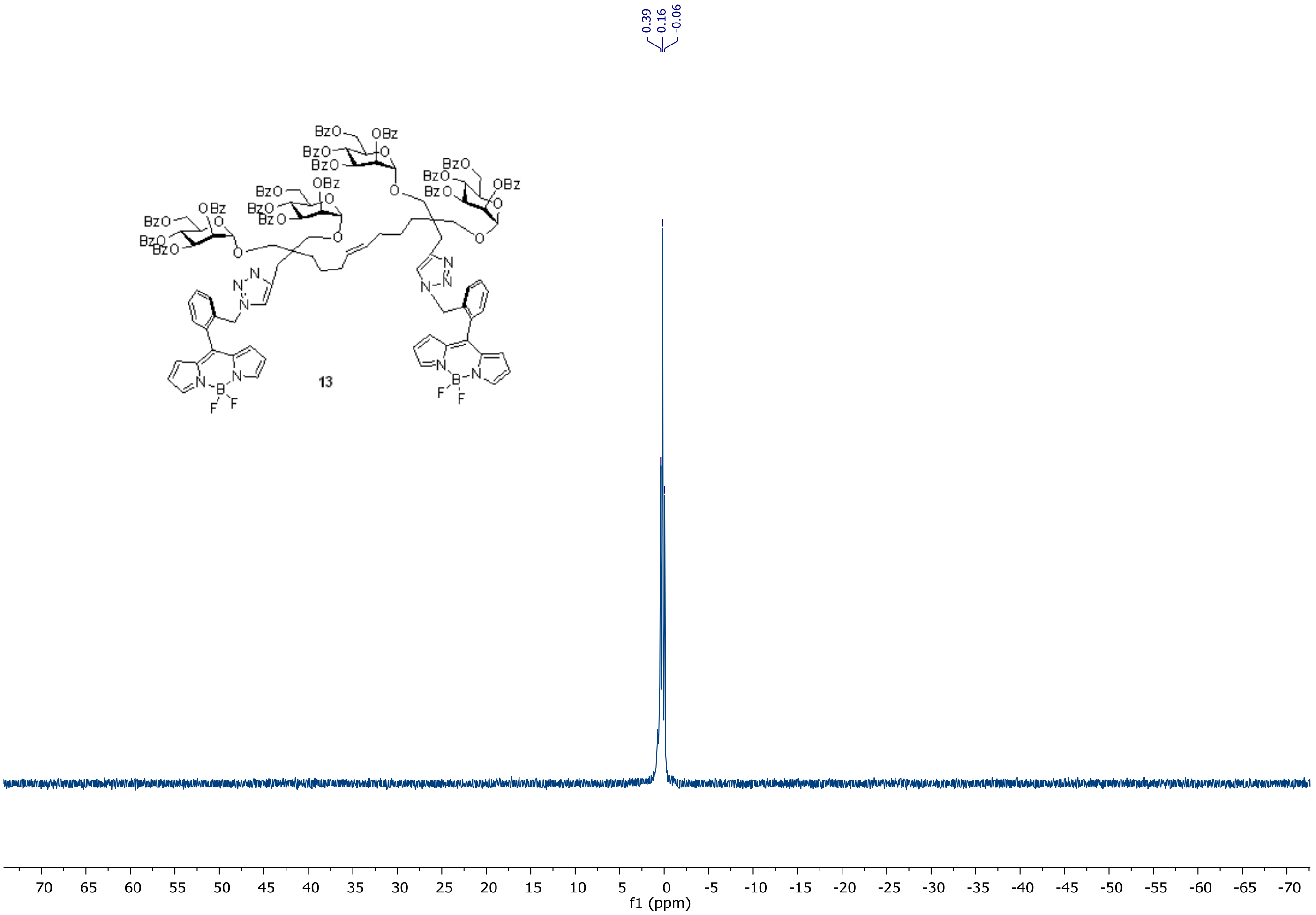

Figure S21. $^{19}$F-NMR spectrum of compound **13**

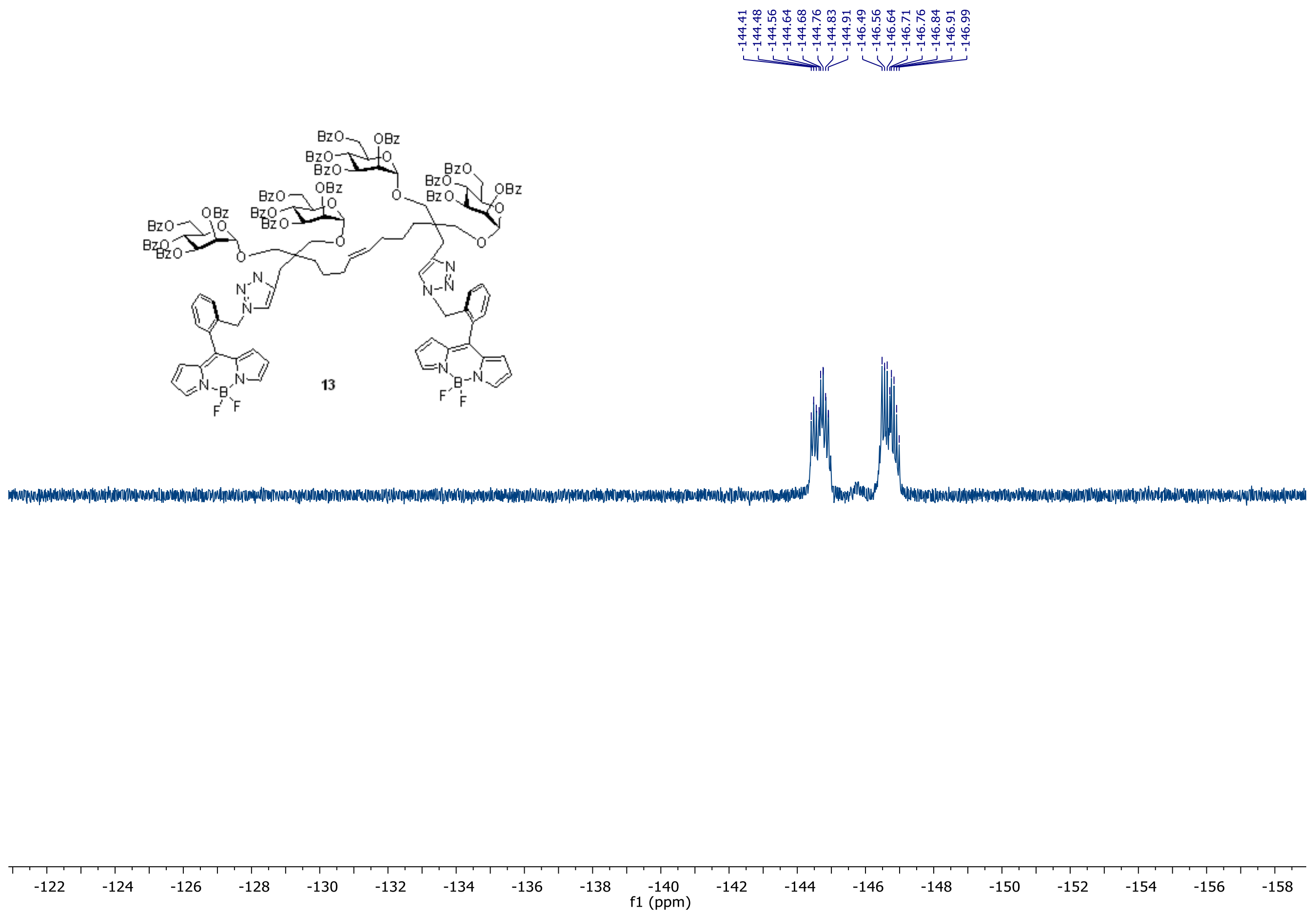


Figure S22. $^{1}$H-NMR spectrum of compound **14**

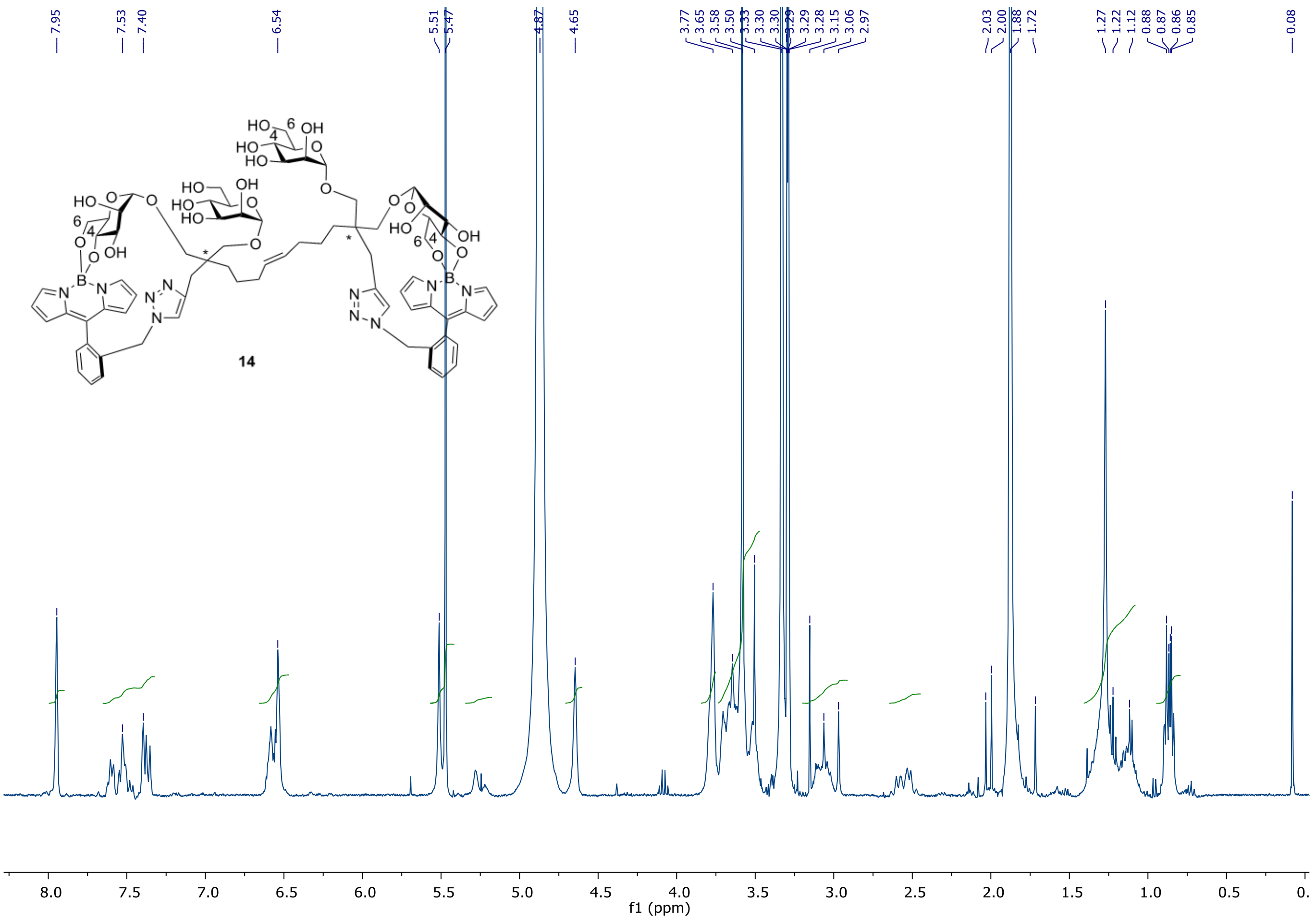

Figure S23. $^{13}$C-NMR spectrum of compound **14**

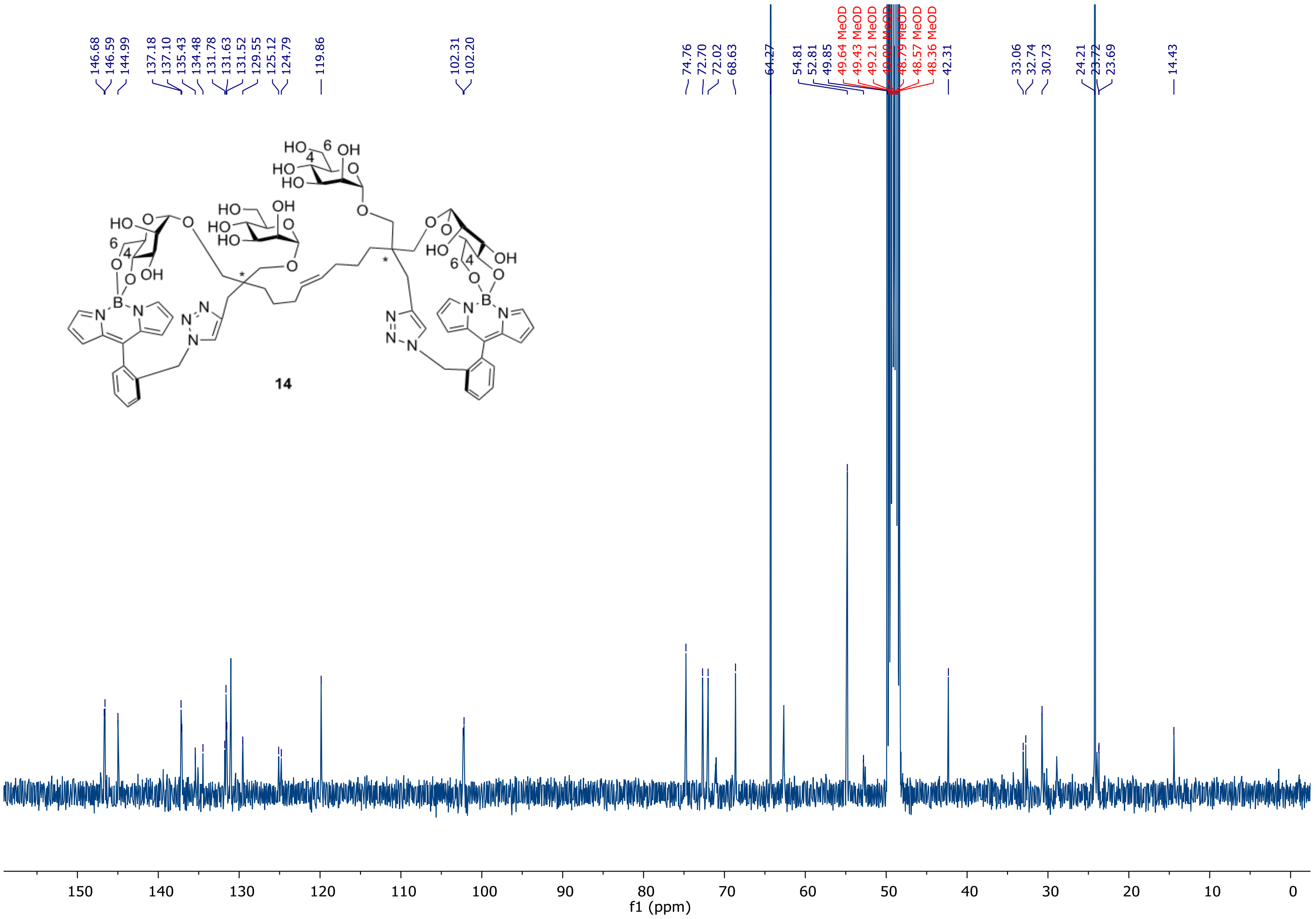


Figure S24. HSQC-NMR spectrum of compound **14**

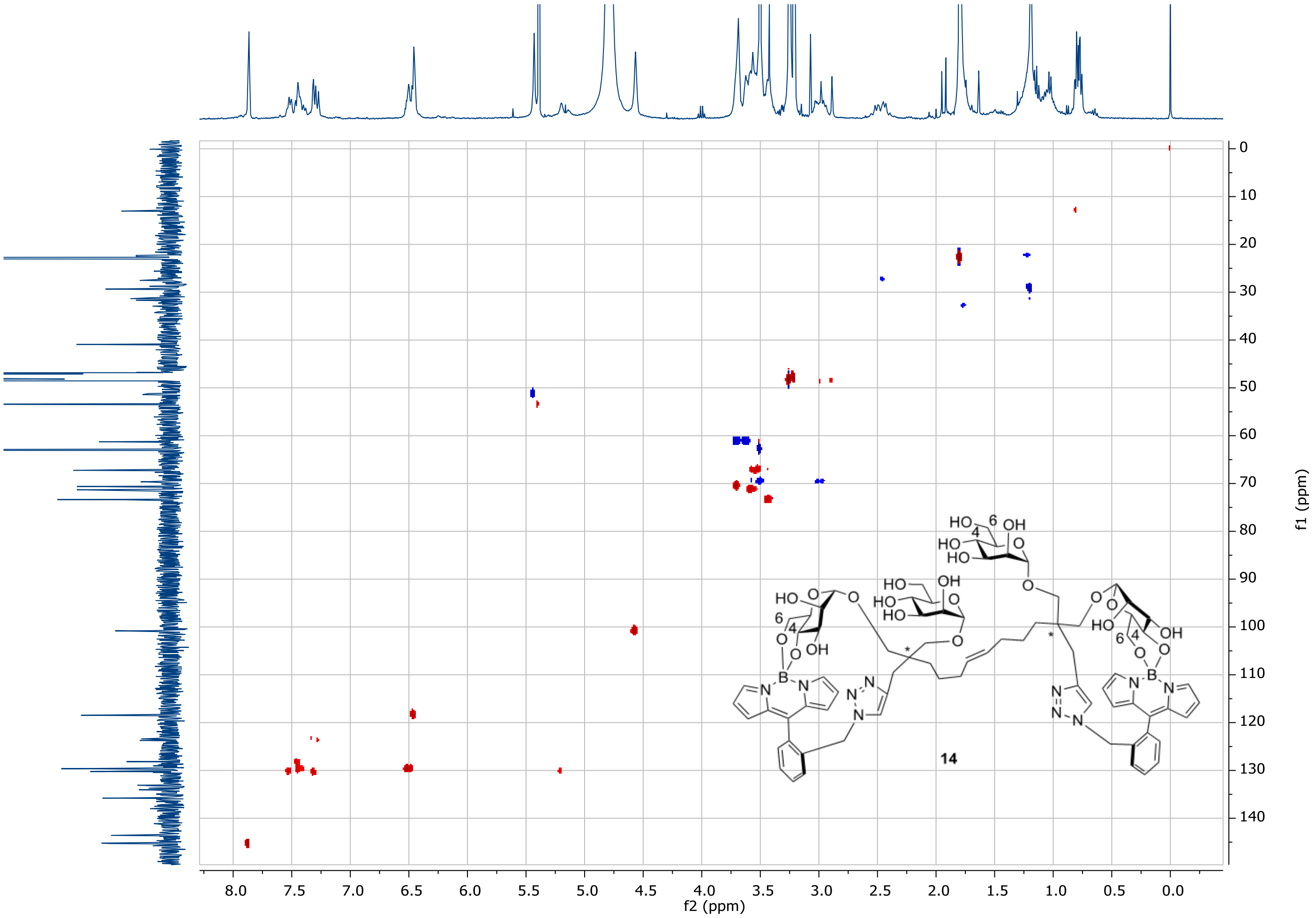

Figure S25. $^{11}$B-NMR spectrum of compound **14**

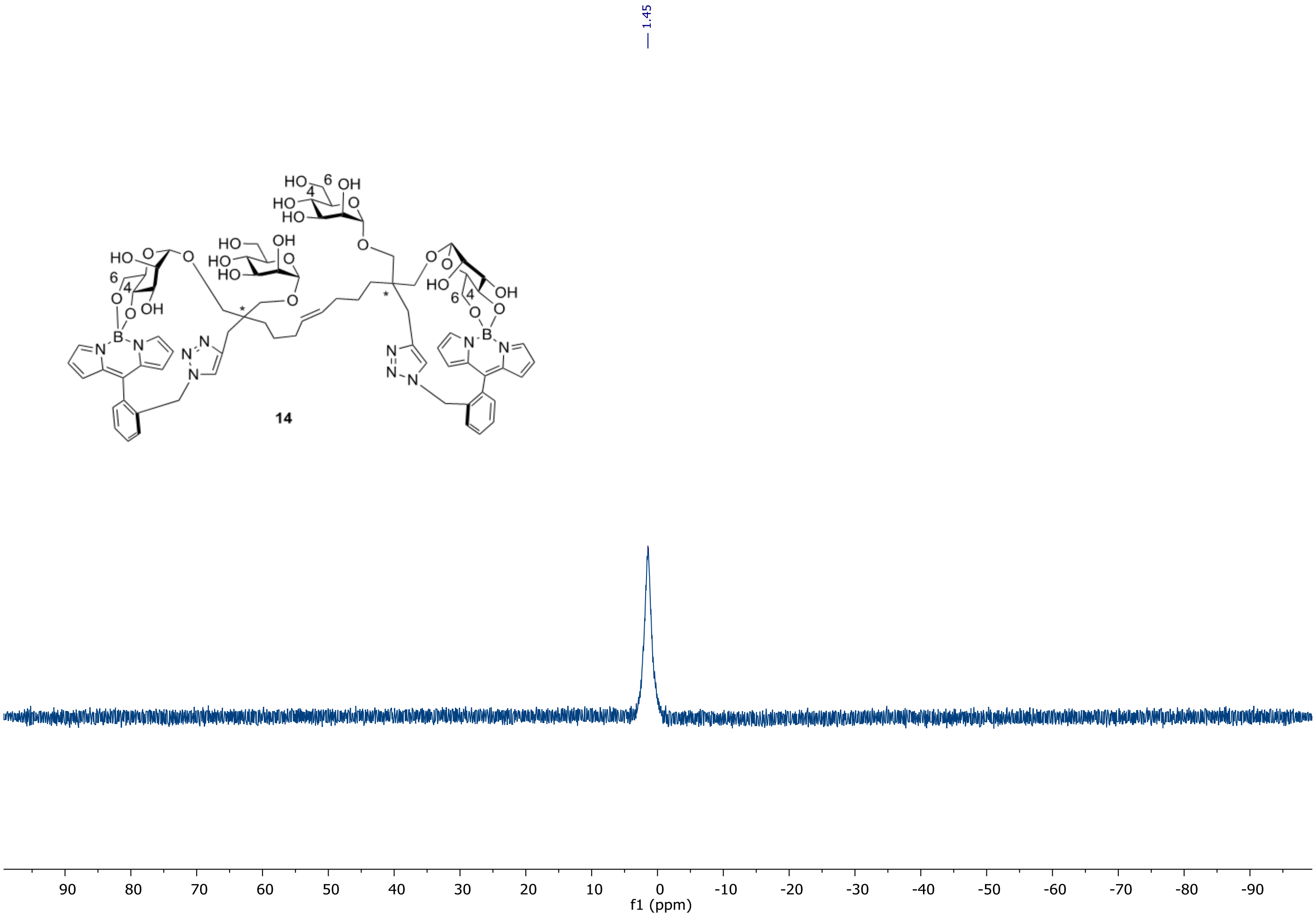


Figure S26. $^{19}$F-NMR spectrum of compound **14**

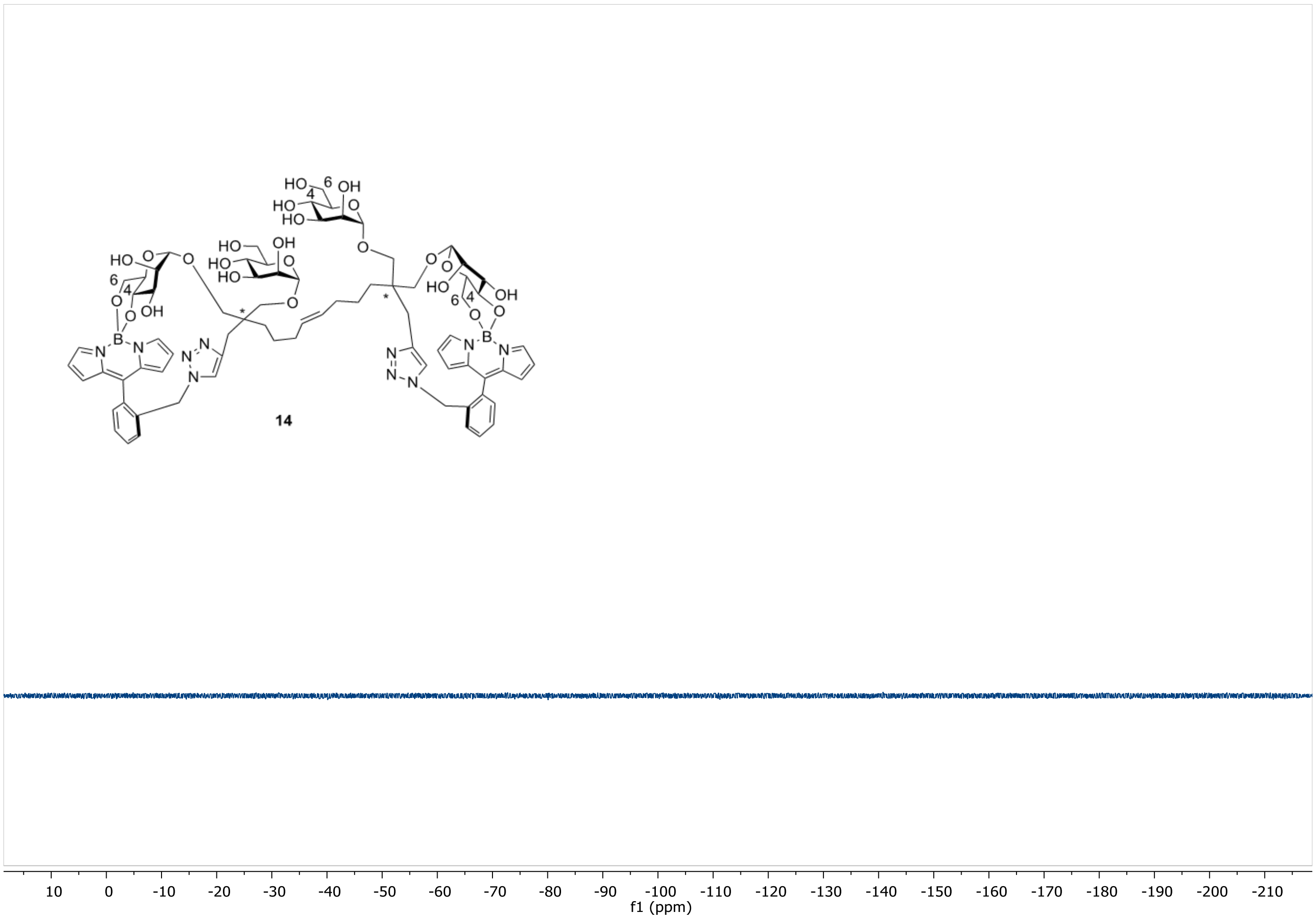

Supplement: Supplementary file 1 [file molecules-24-02050-s001.pdf]
